# Proteomics Coupled with Metabolite and Cell Wall Profiling Reveal Metabolic Processes of a Developing Rice Stem Internode

**DOI:** 10.3389/fpls.2017.01134

**Published:** 2017-07-13

**Authors:** Fan Lin, Brad J. Williams, Padmavathi A. V. Thangella, Adam Ladak, Athena A. Schepmoes, Hernando J. Olivos, Kangmei Zhao, Stephen J. Callister, Laura E. Bartley

**Affiliations:** ^1^Department of Microbiology and Plant Biology, University of Oklahoma Norman, OK, United States; ^2^Waters Corporation Beverly, MA, United States; ^3^Biological Sciences Division, Pacific Northwest National Laboratory Richland, WA, United States

**Keywords:** stem, development, cell wall, proteome, metabolite, phosphoprotein, *Oryza sativa* L., culm

## Abstract

Internodes of grass stems function in mechanical support, transport, and, in some species, are a major sink organ for carbon in the form of cell wall polymers. This study reports cell wall composition, proteomic, and metabolite analyses of the rice elongating internode. Cellulose, lignin, and xylose increase as a percentage of cell wall material along eight segments of the second rice internode (internode II) at booting stage, from the younger to the older internode segments, indicating active cell wall synthesis. Liquid-chromatography tandem mass spectrometry (LC-MS/MS) of trypsin-digested proteins from this internode at booting reveals 2,547 proteins with at least two unique peptides in two biological replicates. The dataset includes many glycosyltransferases, acyltransferases, glycosyl hydrolases, cell wall-localized proteins, and protein kinases that have or may have functions in cell wall biosynthesis or remodeling. Phospho-enrichment of internode II peptides identified 21 unique phosphopeptides belonging to 20 phosphoproteins including a leucine rich repeat-III family receptor like kinase. GO over-representation and KEGG pathway analyses highlight the abundances of proteins involved in biosynthetic processes, especially the synthesis of secondary metabolites such as phenylpropanoids and flavonoids. LC-MS/MS of hot methanol-extracted secondary metabolites from internode II at four stages (booting/elongation, early mature, mature, and post mature) indicates that internode secondary metabolites are distinct from those of roots and leaves, and differ across stem maturation. This work fills a void of in-depth proteomics and metabolomics data for grass stems, specifically for rice, and provides baseline knowledge for more detailed studies of cell wall synthesis and other biological processes characteristic of internode development, toward improving grass agronomic properties.

## Introduction

The grass family, Poaceae, includes the cereal crops and represents one of the most wide-spread plant taxonomic groups in terrestrial ecosystems (Kellogg, 2001). Grass stems, also known as culms, mechanically support reproductive structures, transport nutrients, and act as structural and non-structural carbohydrate storage organs (Moldenhauer et al., 2013). In rice, stem internodes rapidly elongate at the beginning of the reproductive stage (Bosch et al., 2011; Slewinski, 2012). Many important biological processes including cell division, cell wall synthesis, and cell wall remodeling occur during stem development (Bosch et al., 2011; Cui et al., 2012). Among these processes, the change in cell walls is especially important for stem mechanical properties (Gritsch and Murphy, 2005; Wang et al., 2012). Also, the developmental stage of stems influences susceptibility to pests and pathogens (Viajante and Heinrichs, 1987; Bandong and Litsinger, 2005).

Though overall culm development is acropetal, with younger internodes at the top away from the roots, each grass internode exhibits basipetal development. The intercalary meristem, located at the bottom of each internode, undergoes cell division to produce new cells that only have primary cell walls, which in grasses are rich in cellulose and matrix polysaccharides such as arabinoxylan, mixed linkage glucan, pectins, and mannan (Carpita, 1996). The new cells elongate and gradually mature, forming secondary walls toward the apex of each internode (Kende et al., 1998). Associated with this developmental progression, or gradient, is a change in cell wall composition dominated by accumulation of secondary cell walls, which are deposited between the plasma membrane and primary walls in some cell types, such as fibers and sclerenchyma cells. As with those of dicotyledonous plants, secondary cell walls of grasses usually contain multiple, differentially oriented layers of cellulose microfibers (Crow and Murphy, 2000; Thomas et al., 2015). The secondary walls are also layered or impregnated with covalently crosslinked, phenylpropanoid-derived lignin polymer and have greater strength, but lower extensibility, relative to primary walls (Cosgrove and Jarvis, 2012). Indeed, in maize, secondary cell wall components such as cellulose, lignin, and a major grass hemicellulose, arabinoxylan, increase from the bottom to the top segment of each internode, while other cell wall components decrease (Zhang et al., 2014). These changes are associated with altered abundance of transcripts that encode cell wall synthesis enzymes, cell wall remodeling enzymes, and associated regulatory proteins (Zhang et al., 2014).

Many proteins function in cell wall synthesis and remodeling, or regulate the amounts, localization, or activity of cell wall enzymes. Members of several glycosyltransferase (GT) families, including GT2, GT43, GT47, and GT61, synthesize cellulose and hemicellulose in grasses (Scheible and Pauly, 2004; Scheller and Ulvskov, 2010). Enzymes in the phenylpropanoid pathway synthesize lignin precursors and hydroxycinnamic acids (HCAs). The latter are incorporated into lignin or polysaccharides by so-called BAHD acyl-CoA acyltransferases (ATs) (Withers et al., 2012; Bartley et al., 2013; Molinari et al., 2013; Petrik et al., 2014; Buanafina et al., 2016; Karlen et al., 2016; Lin et al., 2016; Sibout et al., 2016). To date, all cell wall-precursor modifying ATs belong to a subclade of BAHDs dubbed the “Mitchell clade” (Mitchell et al., 2007; Bartley et al., 2013; Karlen et al., 2016). Besides cell wall synthesis proteins, cell-wall-localized glycosyl hydrolases (GHs) function in both degradation and growth of cell walls; extracellular proteins, like arabinogalactan proteins, may function in signaling; and peroxidases and laccases catalyze lignin polymerization (Passardi et al., 2004; Jamet et al., 2006; Albenne et al., 2013; Frankova and Fry, 2013; Cosgrove, 2016). Cell wall enzyme transcript abundance is regulated by transcription factors predominantly in the NAC, MYB, and WRKY families (Gray et al., 2012; Handakumbura and Hazen, 2012; Wang and Dixon, 2012). In addition to transcriptional regulation, some cell wall synthesis enzymes and transcription factors are regulated by phosphorylation (Taylor, 2007; Chen et al., 2010, 2016; Wang J. P. et al., 2015). For example, phosphorylation of Arabidopsis Cellulose Synthase (CES) A1 and CESA3 is important for producing ordered interfaces among cellulose microfibrils and macrofibrils and therefore affects anisotropic elongation of cells (Chen et al., 2010, 2016). In addition, phosphorylation of Arabidopsis CESA7 leads to rapid proteosomal degradation (Taylor, 2007). In an example from pine, phosphorylation by PtMAPK6 of a cell wall-related transcription factor, PtMYB4, enhances transcriptional activation of cell wall synthesis genes (Morse et al., 2009).

Sophisticated manipulation of biosynthetic and regulatory pathways is enabled by an accurate knowledge of the molecules present in a particular biological system. Transcriptome data for internodes of several grasses have recently been reported, including for rice, maize, switchgrass, and *Setaria* (Bosch et al., 2011; Hirano et al., 2013; Shen et al., 2013; Zhang et al., 2014; Martin et al., 2016). However, numerous studies report a poor correlation between transcript and protein abundance, which is likely due to various post-transcriptional regulatory events (Vogel and Marcotte, 2012; Albenne et al., 2013; Haider and Pal, 2013; Walley et al., 2016). For example, fewer than 60% of transcripts are translated into proteins in maize (Walley et al., 2016). The current grass stem proteome data for rice, *Brachypodium distachyon*, sugarcane and bamboo consist of fewer than 600 proteins for a given species (Yang et al., 2006; Cui et al., 2012; Douché et al., 2013; Calderan-Rodrigues et al., 2016). Previous gel-based proteomics studies of rice stems identified fewer than 300 proteins, likely missing many proteins involved in important biological processes (Nozu et al., 2006; Yang et al., 2006). There is a similar dearth of metabolite profiling data for grass stems except for sugarcane, which is highly specialized in soluble carbohydrate storage (Glassop et al., 2007). A catalog of rice stem proteins and metabolites will provide a solid foundation for synthetic biology approaches to enhance stem properties and facilitate comparisons with data from other rice organs (Koller et al., 2002) and across species (Kalluri et al., 2009; Printz et al., 2015).

We report here the abundance of biological components in rice internode II during elongation, including variation in cell wall components, and an in-depth protein catalog and a low-depth phosphopeptide catalog of the entire internode. In addition to known cell wall synthesis enzymes that synthesize lignin and polysaccharides, the presence of several putative acyltransferases and glycosyltransferases imply their function in cell wall synthesis. We also report soluble metabolites for internode II during elongation and three post elongation stages. Among the confidently identified metabolites that change over development, are some that have a role in plant-herbivore or plant-pathogen interactions, such as tricin 7-glucoside and esculetin.

## Results

**Figure 1:**
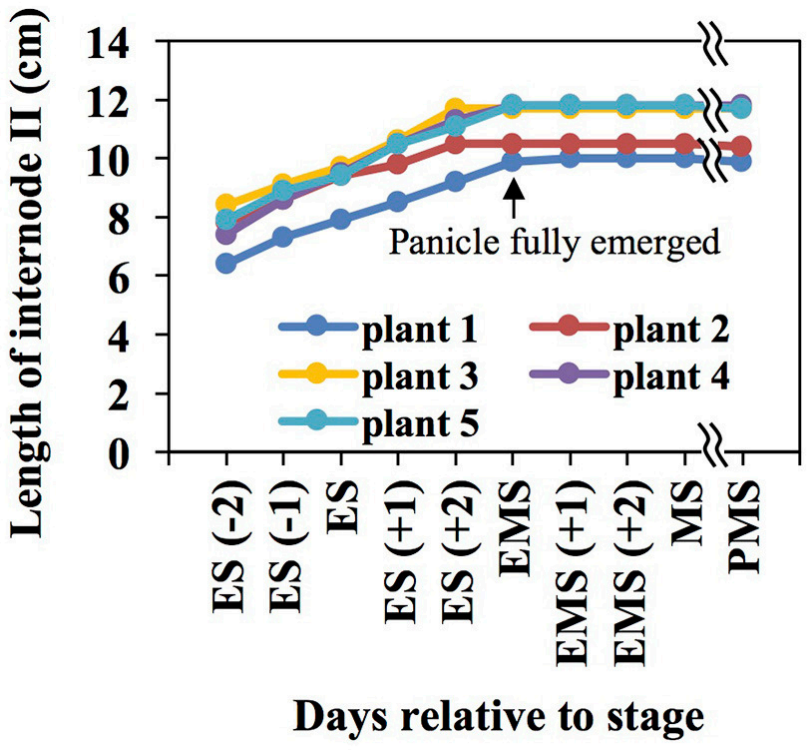
The rice internode II elongates until the panicle fully emerges from the leaf sheath. Internode lengths were measured across four developmental stages: ES, elongating stem, the panicle about to emerge from the flag leaf sheath; EMS, early mature stem, panicle fully emerged from the flag leaf sheath; MS, mature stem, green husks and milky grains; PMS, post mature stem, yellow husks and hard grains. Negative numbers and positive numbers in brackets indicate days before and after a stage, respectively.

During booting, when the panicle approaches emergence from the leaf sheath (BBCH stage 45), internode II of the rice stem undergoes rapid elongation and secondary cell wall development. Internode II is the second internode below the panicle, or the internode immediately below the peduncle (Yamaji and Ma, 2014). Measurement of rice internode II length revealed that it elongates at a relatively constant rate until the panicle fully emerges from the sheath of the flag leaf (Figure 1). This study reports three different datasets collected on rice internode II during elongation and subsequent panicle maturation. First, we report cell wall composition of eight asymmetrical segments of internode II of the elongating stem (ES) during booting. Second, we describe proteomics and phosphoproteomics in whole, unsegmented internode II samples at the same stage. Third, we describe metabolites present in whole internode II samples of the ES and three later stages, ending at seed maturity.

### Cell wall polysaccharides and lignin vary along the elongating rice internode

**Figure 2:**
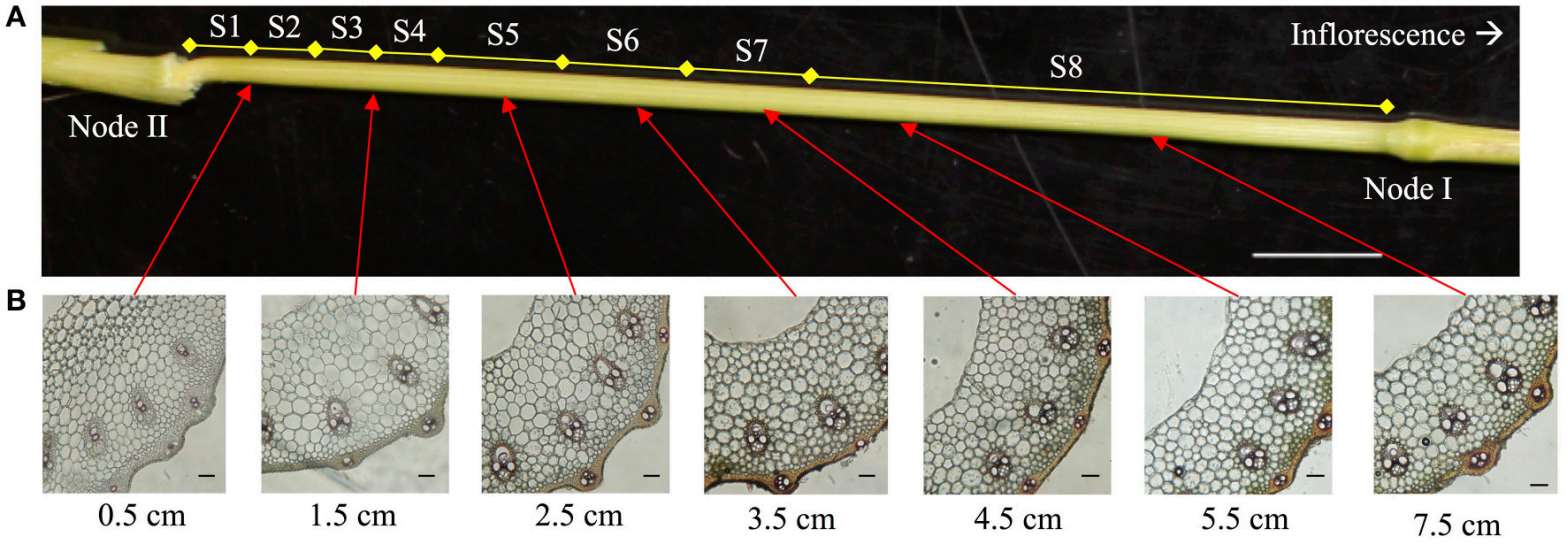
Internode II samples used for cell wall analysis. **(A)** Internode II of elongating stem at booting stage when the panicle is about to emerge. Segments (S1–S8) used for cell wall analysis are indicated. Scale bar is 1 cm **(B)** phloroglucinol stain of cross-sections taken from the bottom (left) of the elongating internode to the top (right) indicate the gradual accumulation of lignin. The labels below each photo indicate the distance from node II. Scale bars are 100 μm.

**Figure 3:**
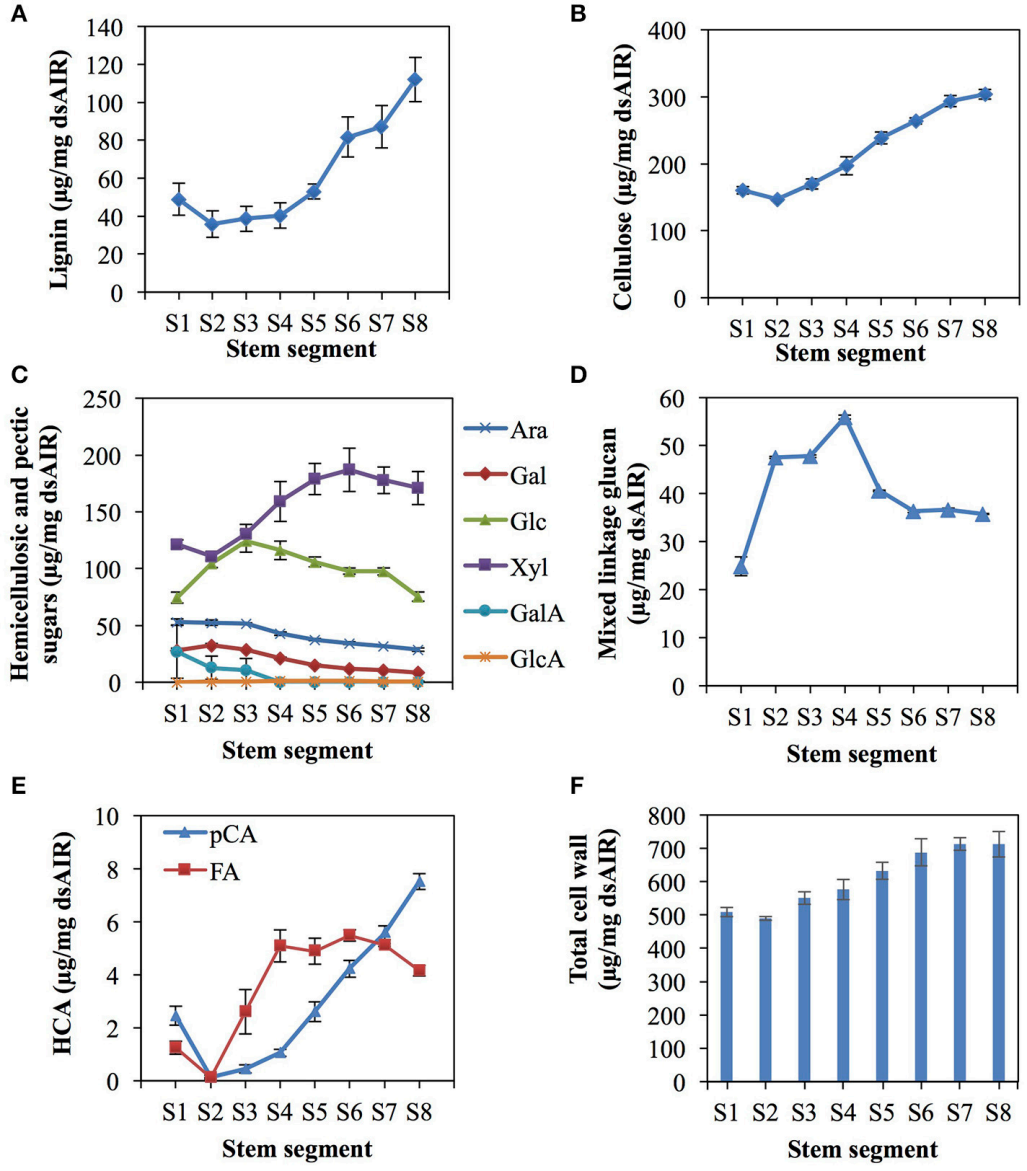
Cell wall composition changes along the rice elongating internode II. The internode was divided into eight uneven segments, from the youngest segment (S1) to the oldest (S8) (See Figure 2). Cell wall components are presented as percent weight of de-starched alcohol insoluble residue (dsAIR). Error bars indicate standard errors of three biological replicates for all measures except lignin, which used four replicates. **(A)** Acetyl bromide soluble lignin. **(B)** Cellulose measured by anthrone assay. **(C)** Hemicellulosic and pectic sugars. Ara, arabinose; Gal, galactose; Glc, glucose; Xyl, xylose; GalA, galacturonic acid; GlcA, glucuronic acid. **(D)** Mixed linkage glucan measured by a lichenase-based kit. **(E)** Cell wall-associated hydroxycinnamic acids (HCAs) consisting of ferulic acid (FA) and *p*-coumaric acid (*p*CA). **(F)** The sum of the mass of cell wall components measured excluding the lichenase-based measure of mixed linkage glucan.

Phloroglucinol staining, indicating the presence of lignin, in rice internode II of the ES was more intense in the mature, upper segments than the young, lower segments, especially around the vascular bundles (Figure 2). Based on the phloroglucinol staining, we divided the internode into eight asymmetrical segments (S1–S8, Figure 2), choosing segment boundaries that minimize the change in cell wall content within each segment, except for S1, which includes the intercalary meristem but also mature tissue below the meristem (See Materials and Methods). The analysis revealed that lignin, cellulose, and xylose significantly increase in terms of mass fraction along the elongating rice internode, while other components like arabinose and glucose decrease, or increase and then decrease (Figure 3, Table S1).

Lignin, measured via acetyl bromide solubilization and expressed as a fraction of de-starched alcohol insoluble residues (dsAIR), generally increased from younger to older internode segments (Figure 3A). The change in lignin was not significant from S1–S4 but dramatically increased from S4–S8 (Tukey's range test, *p* < 0.05) (Table S1). The rapid increase in lignin from S4 to S6 was consistent with the phloroglucinol staining from 1.5 to 3.5 cm above the first node.

Cellulose, determined by the anthrone assay after removing hemicellulose, also generally increased from the younger to the older internode segments (Figure 3B, ANOVA, *p* < 0.01). The mass fraction of cellulose increased continuously from S2 to S7 (Table S1) but did not change significantly between S7 and S8. S1 contained slightly higher cellulose content than S2 but the difference was not statistically significant.

Monosaccharide components of hemicelluloses and other cell wall polysaccharides exhibited different patterns of abundance (Figures 3C,D). Except galacturonic acid and glucuronic acid, trifluoroacetic acid (TFA)-released monosaccharides varied significantly across samples (ANOVA, *p* < 0.01 for xylose, arabinose, glucose, galactose, and mixed linkage glucan). Xylose and arabinose monosaccharides originate mostly from glucuronoarabinoxylan, the most abundant grass hemicellulose. Xylose increased from S2 to S6 (Tukey's range test, *p* < 0.05) (Figure 3C, Table S1), but did not change significantly from S1 to S2 and from S6 to S8. Arabinose, did not change significantly from S1 to S3 but decreased continuously from S3 to S8 (Tukey's range test, *p* < 0.05) (Figure 3C). The other major hemicellulose, mixed linkage glucan (MLG), consists entirely of glucose linked via ß-(1-3) and ß-(1-4) bonds. TFA-released glucose mostly comes from MLG, though amorphous cellulose and xyloglucan also contribute. TFA-released glucose peaked at S3 and then gradually decreased. We also measured MLG with a specific lichenase-based assay, which showed a similar, but not identical pattern of abundance compared to the glucose measurement, by peaking instead at S4 (Figure 3D). TFA-released galactose, mainly from arabinogalactan proteins and pectins, decreased significantly from S2 to S8 (Tukey's range test, *p* < 0.05) (Figure 3C).

Among measured components, cell wall hydroxycinnamoyl esters increased significantly and dramatically along the elongating internode. In grass cell walls, these hydroxycinnamic acids are esterified to lignin, arabinoxylan, and possibly other polysaccharides (Buanafina, 2009; Lin et al., 2016), with the majority of ferulate (FA) on arabinose residues of glucuranoarabinoxylan, and the majority of *p*-coumarate (*p*CA) esterified to lignin (Ralph, 2010; Bartley et al., 2013). We observed differences among segments of 56- and 36-fold for *p*CA and FA, respectively (Figure 3E, ANOVA, *p* < 0.01). For example, FA is lowest in S2 at 0.1 μg/mg biomass and rapidly increases to about 5 μg/mg by S4. Both hydroxycinnamoyl esters increased from younger to the older internode segments; however, FA and *p*CA showed different patterns of accumulation (Figure 3E). FA rapidly increased from S2 to S4, but varied little from S4 to S8. In contrast, *p*CA increased continuously from S2 to S8. The steady increase of the FA:Ara and *p*CA:lignin ratios (Table S1) suggest an increase in both types of cell wall modifications across stem development.

As a fraction of alcohol insoluble residue, the total mass of all uniquely measured cell wall components increased from about 500 μg/mg in S1 and S2 segments to about 700 μg/mg in S7 and S8 segments (Figure 3F). That we measured less than one hundred percent of the cell wall may be due to undetected minerals, especially silica, and incomplete hydrolysis.

### Protein catalog of the elongating rice internode

In order to understand the proteins and phosphoproteins available to participate in cell wall changes and other biological processes during rice stem elongation, we measured trypsin-digested peptides from whole ES internode II samples with three LC-MS/MS experiments using different instruments and analysis methods (Table 1). Experiment 1 and Experiment 2 are from the same sample pool divided after trypsin digestion. Experiment 2 was conducted via Waters SYNAPT G2-Si high definition mass spectrometer with ion mobility-assisted data-independent analysis (HDMS^E^) (Waters, 2011). Experiment 3 was collected with a different method and instrument on an independently grown pool of rice ES internode II samples and serves as a biological replicate to validate the proteins identified in Experiment 2. The phosphoproteomics were conducted on another ES internode II sample and the identified phosphopeptides were validated by the identifications in the phosphopeptide fraction in Experiment 3. Samples were compared by aligning the false discovery rates to 4%. The datasets are available through ProteomeXchange.

**Table 1:** Samples and methods for the three proteomics experiments in this study.

	**Experiment 1**	**Experiment 2**	**Experiment 3**
Sample	Elongating internode rep 1^a^	Same as experiment 1^b^	Elongating internode rep 2^a^
Technical replicates	3	Same as experiment 1	4-plex with isobaric labeling
Protein extraction	Tris buffered phenol	Same as experiment 1	NH_4_Ac buffered MeOH/β-mercaptoethanol
Trypsin digestion	In-gel digestion	Same as experiment 1	Buffered protein suspension
Protein fractionation prior to LC-MS/MS	PAGE divided into 5 sections of different apparent molecular weight	Same as experiment 1	Off line fraction collection using high-pH C18 reversed phase LC followed by pooling into 12 fractions
Liquid chromatography	C18 reversed phase	C18 reversed phase	C18 reversed phase
Mass spectrometer	LTQ Orbitrap (Thermo Fisher)	SYNAPT G2-S*i* HDMS (Waters)	Q Exactive Plus (Thermo Fisher)
Data acquisition mode	Data dependent acquisition	Data independent acquisition	Data dependent acquisition
Peptide search	Proteome Discoverer v1.4, SEQUEST algorithm	Progenesis QIP v2.0	MS-GF+ v2017.01.13
Trypsin mis-cleavage	2	1	Not applicable
Peptide mass tolerance (ppm)	25	20	10
Peptides per protein	≥2	≥2	≥2
Peptide false discovery rate	<5%	<4%	<4%

a *Internode II of ES is defined as the elongating internode. Elongating internode replicate (rep) 1 and rep 2 were biologically matched samples, harvested separately*.

b *Experiment 1 and Experiment 2 used the same peptide samples prepared from elongating internode rep 1*.

Experiment 1, Experiment 2, and Experiment 3 identified 848, 2,872, and 11,602 proteins, respectively, with at least 2 unique peptides in whole elongating internode II samples (Figure S1). The peptide samples for Experiment 1 and Experiment 2 represent the same biological sample analyzed with different techniques and demonstrate reproducibility across different platforms and analysis methods. However, since protein coverage of Experiment 1 was relatively low, we have excluded those data from further analysis. Experiment 3, which represents a distinct biological replicate, shared 2,547 proteins with Experiment 2 (Figure S1, Table S2). The proteins commonly identified in the two biological replicates in Experiment 2 and Experiment 3 correspond to 6% of non-transposon genes in the rice genome annotation. About 54% of the 2,547 identified rice proteins have not been previously reported (by 2 or more unique peptides) in the Rice Proteogenomics Database (Figure S2) (Helmy et al., 2012). We therefore have been able to extend the current protein catalog of rice proteins. Subsequent analyses presented here focus exclusively on the 2,547 proteins commonly identified in Experiments 2 and 3 (Table S2).

In addition to reporting on peptide counts and peptide coverage, Experiments 2 and 3 both employed quantitative methodologies. In Experiment 2, the protein abundance was calculated from ion intensity based on the response factor, in ion intensity per fmol, of a known-concentration of an external standard, yeast alcohol dehydrogenase (ADH1_YEAST). For Experiment 2, the average coefficient of variation (CV) among technical replicates was 17% (Table S2). In Experiment 3, the abundance of peptides was quantified with isobaric tagging for relative and absolute quantitation (iTRAQ) and is expressed as arbitrary “peak intensity” based upon the fragment ion containing the isobaric tag. The average CV among technical replicates was 2%. The protein abundance from Experiment 2 and Experiment 3 are significantly (*p* < 0.01) but not highly correlated. The Pearson correlation coefficient and Spearmen correlation coefficient for the two quantitative datasets from the two biological replicates are 0.23 and 0.29, respectively (Figure S3). Quantitation from each method for the 2,547 commonly identified proteins is available in Table S2.

### Proteome gene ontology (GO) and KEGG ontology (KO) analysis

We performed gene ontology (GO) enrichment analysis on cellular component, biological process, and molecular function terms for the proteins detected in the elongating rice internode II (Table S3).

**Figure 4:**
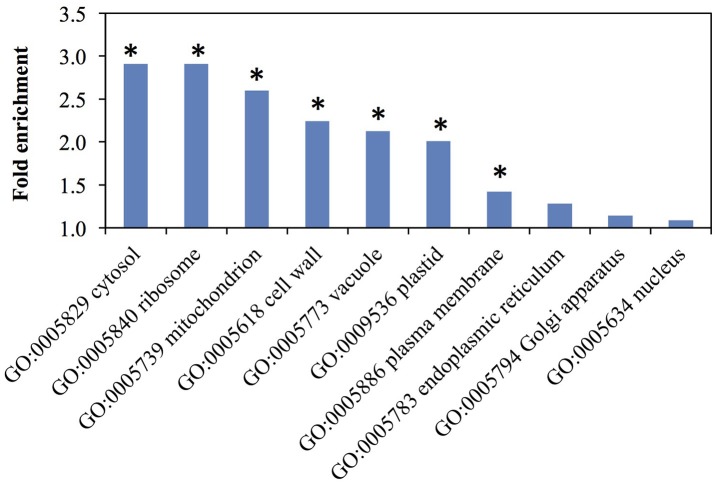
Representation of cellular component Gene Ontology (GO) terms among rice elongating internode proteins. Asterisks indicate over-representation with a *p*-value <0.01 by hypergeometric test. Fold enrichment is the ratio of identified proteins to the expected number of proteins for each GO term.

Cellular Component GO-term enrichment showed some location biases in our dataset (Figure 4 and Table S3) Hypergeometric *p*-values indicate that the GO-terms of cytosol, ribosome, mitochondrion, cell wall, vacuole, plastid, and plasma membrane are over-represented. The GO-terms of endoplasmic reticulum, Golgi apparatus, and nucleus are present at the expected frequencies. These terms cover the cellular fractions where cell wall-related proteins like glycosyltransferases (GTs), phenylpropanoid enzymes, and glycosyl hydrolases (GHs) have been found. Specifically, GTs primarily localize to the Golgi (Nikolovski et al., 2012) but also the plasma membrane (Bashline et al., 2014) and cytosol (Rautengarten et al., 2011); phenylpropanoid enzymes localize to the cytosol and endoplasmic reticulum (Ro et al., 2001; Achnine et al., 2004); and GHs typically localize to cell walls (Chen et al., 2009).

**Figure 5:**
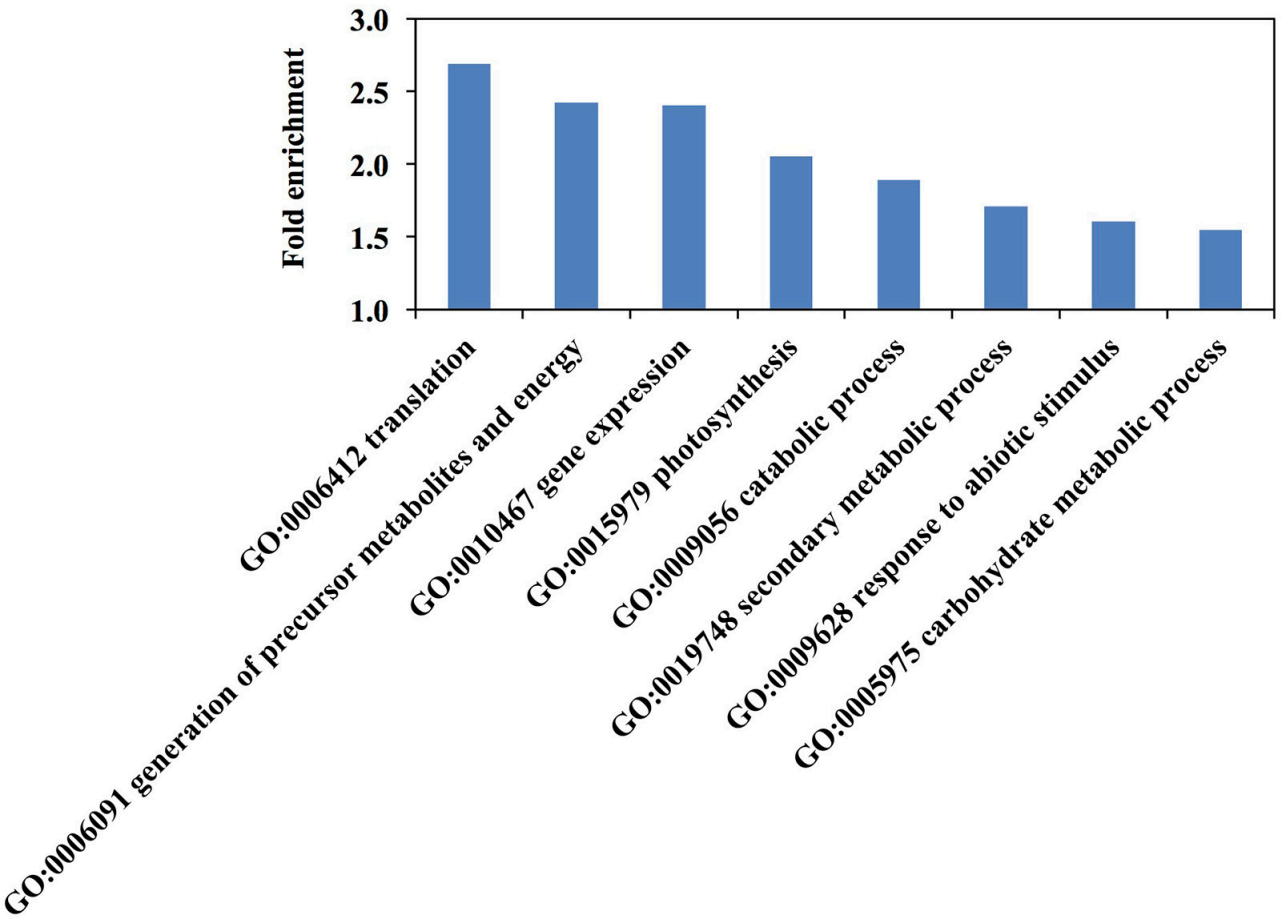
Top over-represented biological process Gene Ontology (GO) terms among rice elongating internode II proteins. Redundant GO terms were removed. All terms are over-represented with a *p*-value <0.01 by hypergeometric test. Fold enrichment is the ratio of identified proteins to the expected number of proteins for each GO term.

Enrichment analysis of biological process and molecular function GO-terms revealed some categories overrepresented in the rice elongating internode II (Figure 5 and Table S3). The most highly overrepresented biological process GO-terms relate mostly to cellular metabolic process such as “cellular macromolecule biosynthetic process” and “cellular biosynthetic process.” “Generation of precursor metabolites and energy” and “secondary metabolic process” were also significantly over-represented in the data (Figure 5).

Since many metabolic processes are overrepresented in the GO analysis, we conducted KEGG pathway mapping to further examine representation of identified proteins in different metabolic pathways (Kanehisa et al., 2016). For all the 2,547 proteins identified, we found 1,003 proteins annotated by 756 KEEG Ontology (KO) terms, including 165 proteins assigned to more than one KO term (Table S4). Among them, 262 proteins, annotated by 167 KO-terms, were in secondary metabolite biosynthesis pathways such as the phenylpropanoid pathway. The 17 proteins in the phenylpropanoid pathway include many of the major enzymes required to synthesize monolignols (Table 2, Figure S4), except ferulate 5-hydroxylase and caffeic acid *O*-methyltransferase.

**Table 2:** Rice phenylpropanoid-active enzymes observed in the elongating internode.

**Locus ID^a^**	**Gene family^b^**	**Peptide count^c^**	**Unique peptides^d^**	**Function or putative function**	**Citation**
LOC_Os02g41630	PTAL	83	50	Putative phenylalanine/tyrosine ammonia-lyase	
LOC_Os05g25640	C4H	3	2	Putative cinnamic acid 4-hydroxylase	
LOC_Os02g08100	4CL	33	21	4CL3, 4-coumarate:CoA ligase	Gui et al., 2011
LOC_Os06g44620	4CL	24	10	4CL4, putative 4-coumarate:CoA ligase	Gui et al., 2011
LOC_Os02g39850	HCT	10	8	HCT2, hydroxycinnamoyl-Coenzyme A shikimate/quinate hydroxycinnamoyltransferase	Kim et al., 2012
LOC_Os04g42250	HCT	6	4	Putative hydroxycinnamoyl-Coenzyme A shikimate/quinate hydroxycinnamoyltransferase	
LOC_Os06g06980	CCoAOMT	14	14	Putative caffeoyl CoA 3-*O*-methyltransferase	
LOC_Os05g41440	C3H	3	2	Putative *p*-coumaroyl shikimate 3′-hydroxylase	
LOC_Os08g34280	CCR	8	5	CCR1, cinnamoyl CoA reductase	Kawasaki et al., 2006
LOC_Os02g09490	CAD	25	25	CAD2, cinnamyl-alcohol dehydrogenase	Zhang et al., 2006
LOC_Os09g23540	CAD	20	4	Putative cinnamyl-alcohol dehydrogenase	
LOC_Os08g02110	POX	9	8	Putative class III plant peroxidase^e^	
LOC_Os02g58720	POX	8	7	Putative class III plant peroxidase^e^	
LOC_Os06g46799	POX	5	4	Putative class III plant peroxidase^e^	
LOC_Os09g29490	POX	5	4	Putative class III plant peroxidase^e^	
LOC_Os01g36240	POX	3	2	Putative class III plant peroxidase^e^	
LOC_Os01g40860	REF	14	8	Putative sinapaldehyde dehydrogenase	

a *Rice Genome Annotation Project (Version 7) (Ouyang et al., 2007)*.

b *Gene families involved in phenylpropanoid biosynthesis. PTAL, phenylalanine/tyrosine ammonia-lyase; C4H, cinnamic acid 4-hydroxylase; 4CL, 4-coumarate:CoA ligase; HCT, hydroxycinnamoyl-Coenzyme A shikimate/quinate hydroxycinnamoyltransferase; CCoAOMT, caffeoyl CoA 3-O-methyltransferase; C3H, p-coumaroyl shikimate 3′-hydroxylase; CCR, cinnamoyl-CoA reductase; CAD, cinnamyl alcohol dehydrogenase; POX, peroxidase; REF, reduced epidermal fluorescence*.

c *Total number of peptides identified for a protein including those that match with other proteins*.

d *The number of unique peptides private to a protein*.

e *Class III peroxidases belong to one of the more than 60 classes of peroxidases as described in the PeroxiBase (updated May 2015, http://peroxibase.toulouse.inra.fr/). Class I-III peroxidases are found only in plants*.

### Cell wall synthesis and remodeling proteins, and extracellular proteins

We hypothesize that several of the identified proteins function in cell wall synthesis within the elongating rice internode II. We examined the GT2, GT43, GT47, GT61, and AT clades that contain known grass cell wall synthesis proteins. We identified two GT2, one GT47, one GT61, and six Mitchell clade-AT proteins (Table 3). GT61-III-1, a GT61 that may synthesizes xylan substituents and OsIRX10, a GT47 that synthesizes the xylan backbone (Chen et al., 2013) were present. In addition to proteins unambiguously identified in both Experiment 2 and 3, the GT61-OsXAX1 (LOC_Os02g22380) (Chiniquy et al., 2012) and GT61-IV-1 (LOC_Os06g27560) were unambiguously identified in Experiment 3 even though they could not be distinguished from each other based on the detected peptide sequences in Experiment 2. The proteins include four hydroxycinnamoyl CoA acyltransferases implicated by biochemical and genetic studies in cell wall modification and two putative feruloyl CoA transferases that have not been experimentally examined (Table 3).

**Table 3:** Rice cell wall-related acyltransferases and glycosyltransferases observed in the elongating internode.

**Locus ID^a^**	**Protein^b^**	**Peptide count^c^**	**Unique peptides^d^**	**Function or putative function**	**Citation**
LOC_Os01g09010	OsAT9	27	22	Candidate feruloyl coenzyme A transferase involved in xylan synthesis	Lin et al., 2016
LOC_Os01g42880	OsAT1	26	22	Ortholog of a putative feruloyl coenzyme A transferase involved in cell wall modification in Brachypodium.	Buanafina et al., 2016
LOC_Os01g42870	OsAT2	11	10	Ortholog of a candidate feruloyl coenzyme A transferase	Molinari et al., 2013
LOC_Os05g04584	OsAT3	11	9	Ortholog of Brachypodiun *p*-coumarate monolignol transferase	Petrik et al., 2014; Sibout et al., 2016
LOC_Os06g39390	OsAT10	9	9	Putative *p*-coumaroyl coenzyme A transferase involved in arabinoxylan modification	Bartley et al., 2013
LOC_Os01g18744	PMT/OsAT4	5	5	*p*-coumarate monolignol transferase	Withers et al., 2012; Petrik et al., 2014
LOC_Os05g08370	GT2-CESA1	5	5	Cellulose synthase	
LOC_Os03g60939	GT2	2	2	Unknown	
LOC_Os01g70200	GT47-OsIRX10	2	2	Involved in xylan backbone synthesis. *Osirx10* mutants possess a short internode phenotype.	Chen et al., 2013
LOC_Os02g22190	GT61-III-1	4	2	Unknown, high expression in root and stem at transcript level	

a *Rice Genome Annotation Project (Version 7) (Ouyang et al., 2007)*.

b *Acyltransferases (AT) and glycosyltransferases (GTs) involved in polysaccharide biosynthesis. PMT, p-coumaroyl-CoA:monolignol transferase; CESA, cellulose synthase A; IRX, irregular xylem. GT61 subclade III and VI were defined in Chiniquy et al. (2012)*.

c *Total number of peptides identified for a protein including those that match with other proteins*.

d *The number of unique peptides private to a protein*.

**Figure 6:**
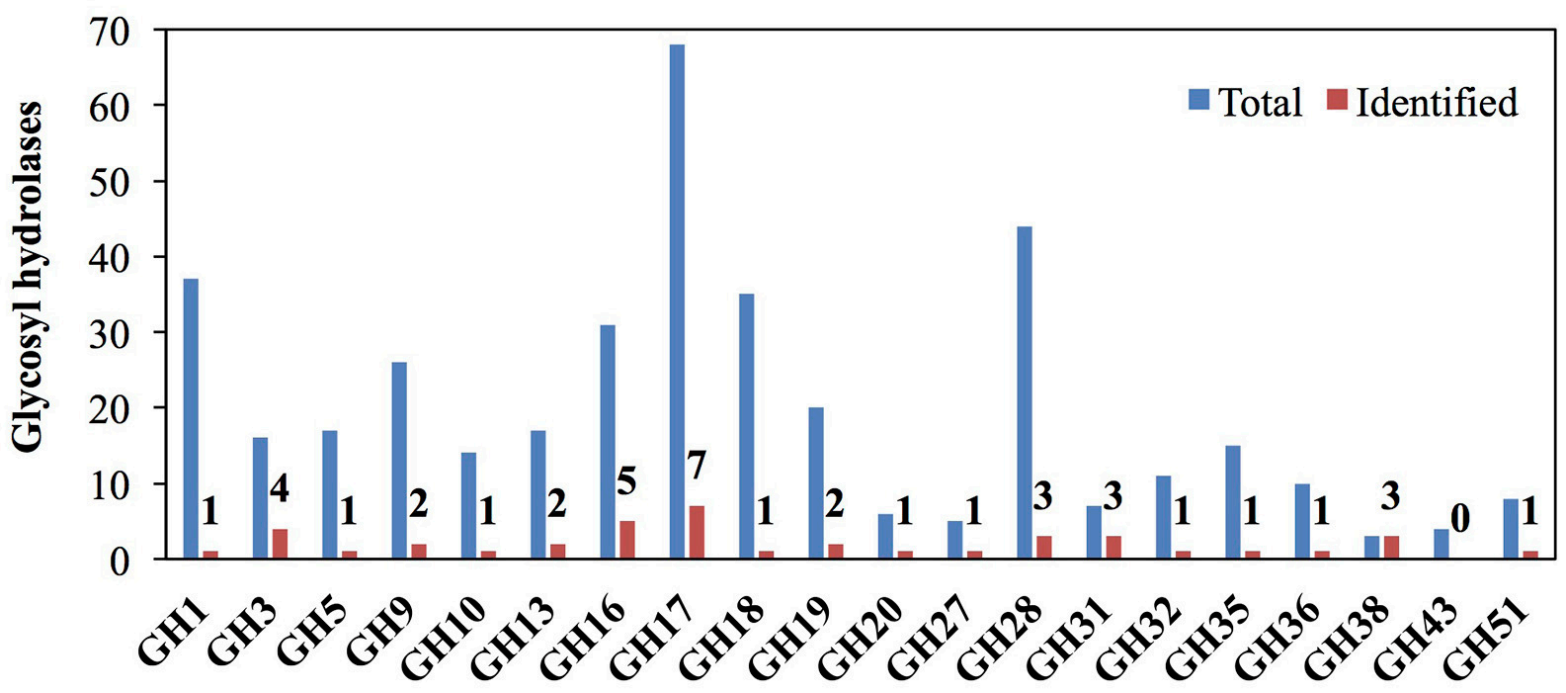
Representation of glycosyl hydrolase (GH) families that may modify or degrade cell walls among rice internode II proteins. Red bars and numbers indicate the GH proteins identified in this study. Blue bars indicate the total number of GH proteins in rice GH database. GH families that modify plant cell wall components, localize to the cell wall, or have much greater numbers in grasses compared to dicots are included.

Glycosyl hydrolases (GHs) are a major class of cell wall remodeling enzyme that modify or degrade the backbone or substituents of polysaccharides (Minic, 2008) and can influence developmental processes such as lateral root emergence, leaf abscission, xylem differentiation, and the mechanical stress response of stems (Agusti et al., 2012; Lewis et al., 2013; Verhertbruggen et al., 2013; Xie et al., 2013; Yu et al., 2013). However, due to the huge number of GHs in plant genomes, only a small proportion of GHs have been functionally characterized. Among the GH families that we examined, enzymes from the GH1, GH3, GH5, GH9, GH13, GH16, GH17, GH18, GH19, GH20, GH27, GH28, GH31, GH38, and GH51 families include extracellular proteins previously reported in rice cell wall proteomics studies that are more likely to have direct functions in cell wall remodeling (Jung et al., 2008; Chen et al., 2009; Cho et al., 2009). In addition, enzymes from the GH1, GH9, GH10, GH17, GH18, GH19, GH28, GH32, GH36, and GH51 families are expanded in grasses and may function particularly on grass-abundant cell wall components, such as arabinoxylans and mixed-linkage glucan (Sharma et al., 2013). We identified 41 proteins in these families (Figure 6 and Table S5) and more than three proteins from each of the following families: GH3, GH16, GH17, GH28, GH31, and GH38 (Figure 6).

WallProtDB constitutes a collection of many extracellular proteins from targeted cell wall proteomics experiments (San Clemente and Jamet, 2015). Fifty-one proteins from elongating rice internodes are represented in WallProtDB. The two most abundant categories are GHs and cell wall-localized type III peroxidases, the latter of which may catalyze polymerization of monolignonols (Passardi et al., 2004) (Figure S5 and Table S6). Besides these two major groups, we also identified nine proteases, four expansins that may function in cell wall-loosening, three leucine-rich repeat proteins that may participate in plant defense, and two fasciclin-like arabinogalactan proteins implicated in plant development and stress-responses (Cosgrove, 2000; Johnson et al., 2003).

### Kinases and transcription factors

Cell wall biosynthesis and other stem developmental processes are regulated by numerous protein kinases and transcription factors (Handakumbura and Hazen, 2012; Wang J. P. et al., 2015). From the rice protein kinase database (Dardick et al., 2007) and the rice TF database, we identified 30 protein kinases, including both receptor-like and cytoplasmic kinases (Table S7), and 46 putative transcription factors (Table S8).

**Figure 7:**
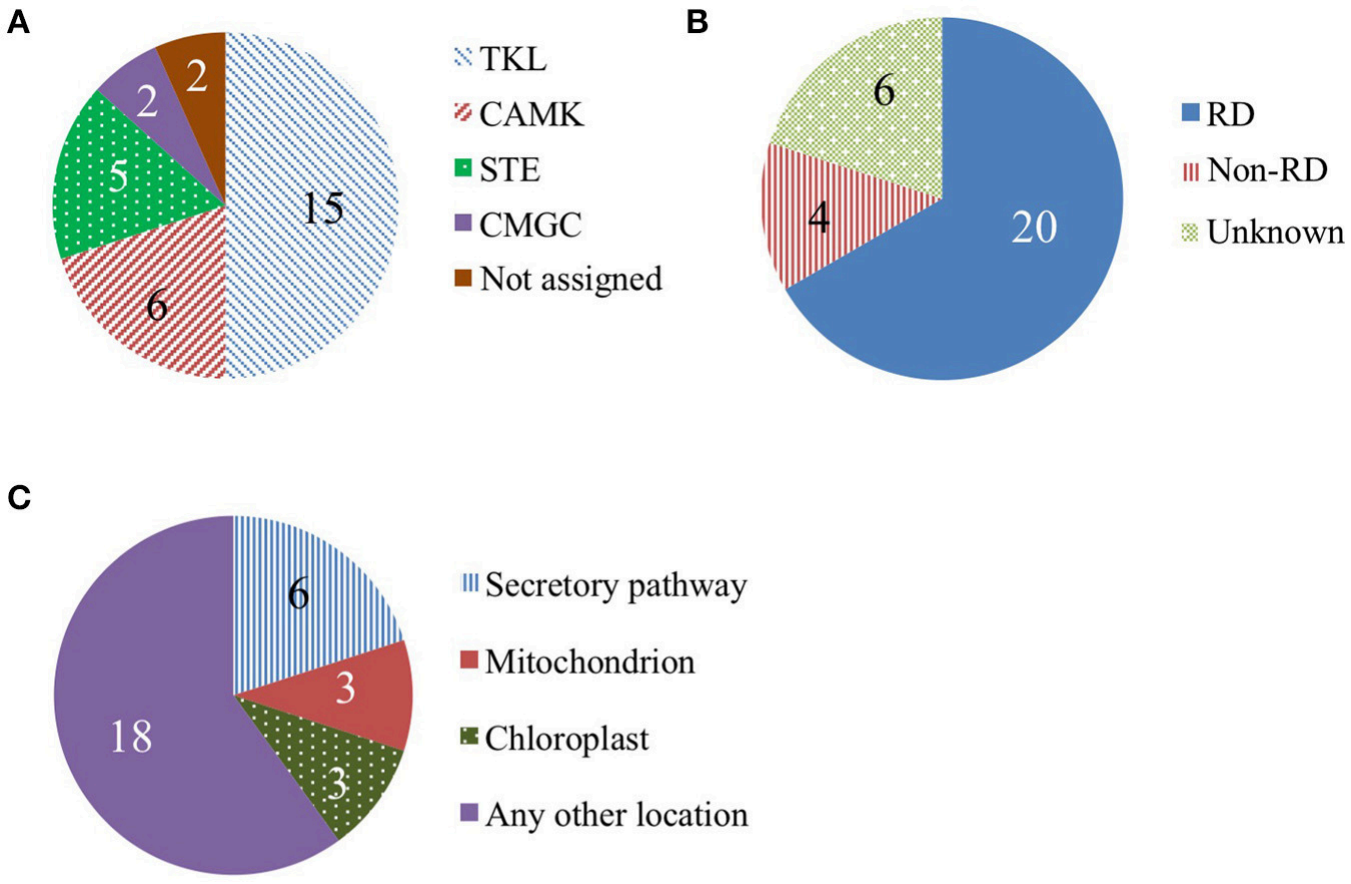
Protein kinases in the rice elongating internode. **(A)** Kinase phylogenetic groups. The TKL (Tyr kinase-like) group includes mixed lineage kinases, transforming growth factor-β receptor kinases, and Raf kinases. The CAMK group consists of calcium/calmodulin-dependent protein kinases. The STE group includes homologs of yeast sterile 7, sterile 11, and sterile 20 kinases. The CMGC group includes CDK, MAPK, GSK3, and CLK kinases. **(B)** Kinase categories based on a conserved kinase RD motif. Kinases containing this motif are RD kinases while non-RD kinases lack the motif. **(C)** Predicted subcellular localization of kinases.

**Figure 8:**
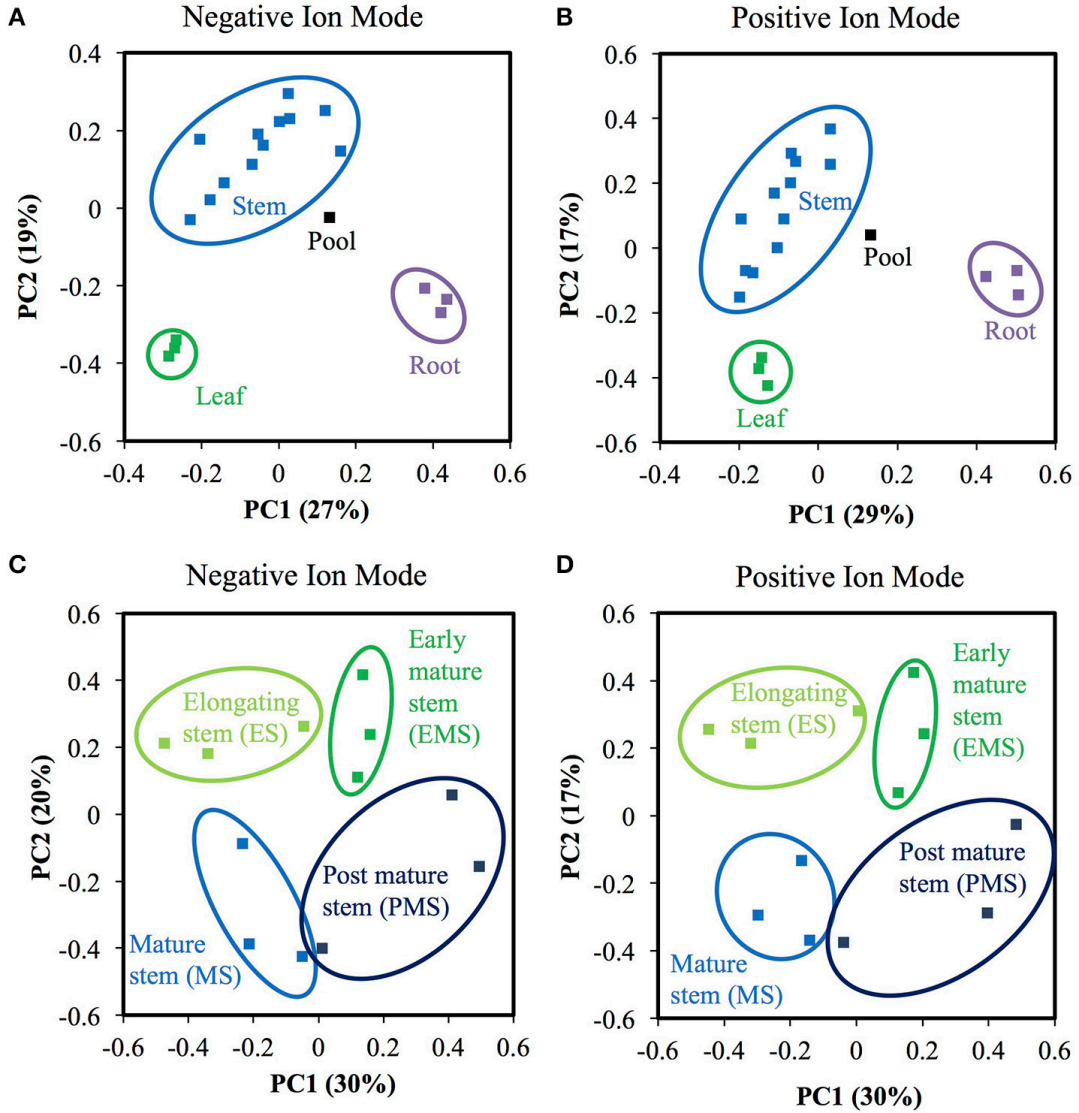
Methanol extracted metabolite profiles differ among rice organs and internode II developmental stages. **(A,B)** Principal component analysis (PCA) of samples from different organs. Leaf samples are represented by green symbols, root are purple, stem are blue. The pool sample is black. **(A)** Negative ion mode. **(B)** Positive ion mode. **(C,D)** PCA of stem samples of different developmental stages. Light green, green, blue, and dark blue represent elongating stem internode (ES), early mature stem internode (EMS), mature stage stem internode (MS), and post mature stem internode (PMS), respectively. **(C)** Negative ion mode. **(D)** Positive ion mode.

We categorized detected protein kinases based on phylogeny, presence of a conserved kinase motif, and predicted subcellular localization (Figure 7). In the literature, protein kinases with cell wall phenotypes or that regulate cell wall genes belong to the following three groups: TKL (Tyr-kinase-like) kinases, CMGC (CDK, MAPK, GSK3, and CLK) kinases, and CAMK (calcium/calmodulin-dependent) kinases (Morse et al., 2009; Oh et al., 2011; Matschi et al., 2013). We found evidence of the presence of 15 TKL kinases, including four from the LRR receptor-like kinase family. The two CMGC kinases identified were both from the MAPK family. The six CAM kinases identified were all calcium-dependent kinases. We also checked if the identified kinases contain a conserved arginine in the RD-motif in kinase subdomain VI. The absence of this motif (i.e., non-RD) correlates with functioning in pathogen response (Dardick and Ronald, 2006). We found 20 RD kinases and only 4 non-RD kinases (Figure 7B). Kinases that regulate plant development and cell wall biosynthesis are often localized to the plasma membrane (Park et al., 2001; Hematy et al., 2007; Oh et al., 2011). Six of the 30 identified proteins are predicted to be secreted and could be plasma membrane-localized (Figure 8C).

As phosphorylation mediated by protein kinases is an important regulatory mechanism for plant development and cell wall synthesis (Park et al., 2001; Hematy et al., 2007; Oh et al., 2011), we conducted a small phosphoproteomics experiment to explore phosphoproteins in the elongating internode. The identified phosphopeptides were further confirmed by their presence in the phosphoprotein-enriched fraction in Experiment 3. In both experiments, we identified a common set of 21 unique phosphopeptides from 20 phosphoproteins (Table S9). Among them, nine were phosphoproteins not reported previously in P3DB, including an LRR-III family receptor like kinase (LOC_Os03g12250).

We identified 46 transcription factors in the proteomics dataset. The C3H family was most common (Figure S6). We also identified a few proteins in cell wall-related or cell division-related transcription factor families: CDC5 (LOC_Os04g28090) from the R3-MYB family and NAC2 (LOC_Os08g06140) from the NAC family (Feller et al., 2011; Wang H. et al., 2015).

### Metabolite profiles of the elongating internode, mature internodes, leaf and root

The proteomics data confirmed the importance of secondary metabolic processes in the internode II of ES, thus, we also analyzed methanol-soluble secondary metabolites, including phenylpropanoids, isolated from different developmental stages of rice internode II. We collected internode II at the following four stages: the same elongating stem (ES) internode as the proteomics experiments, the early mature stem (EMS) internode at flowering, the mature stem (MS) internode at grain filling, and the post mature stem (PMS) internode after seed maturity (Figure 6). For comparison, we also extracted metabolites from mature rice leaves and roots. We analyzed three biological replicates by LC-MS/MS with technical triplication. We reproducibly detected a total of 11,929 and 8,521 metabolite ions across replicate injections of any sample, in negative and positive ionization mode, respectively. (See Materials and Methods; Tables S10, S11). Of these, we tentatively annotated 22 negative ions and 53 positive ions based on mass, isotope similarity, and theoretical fragmentation. We used commercial standards to confirm the MS/MS fragmentation pattern and retention time of a phenylpropanoid, *p*CA, and a flavonoid, apigenin.

**Figure 9:**
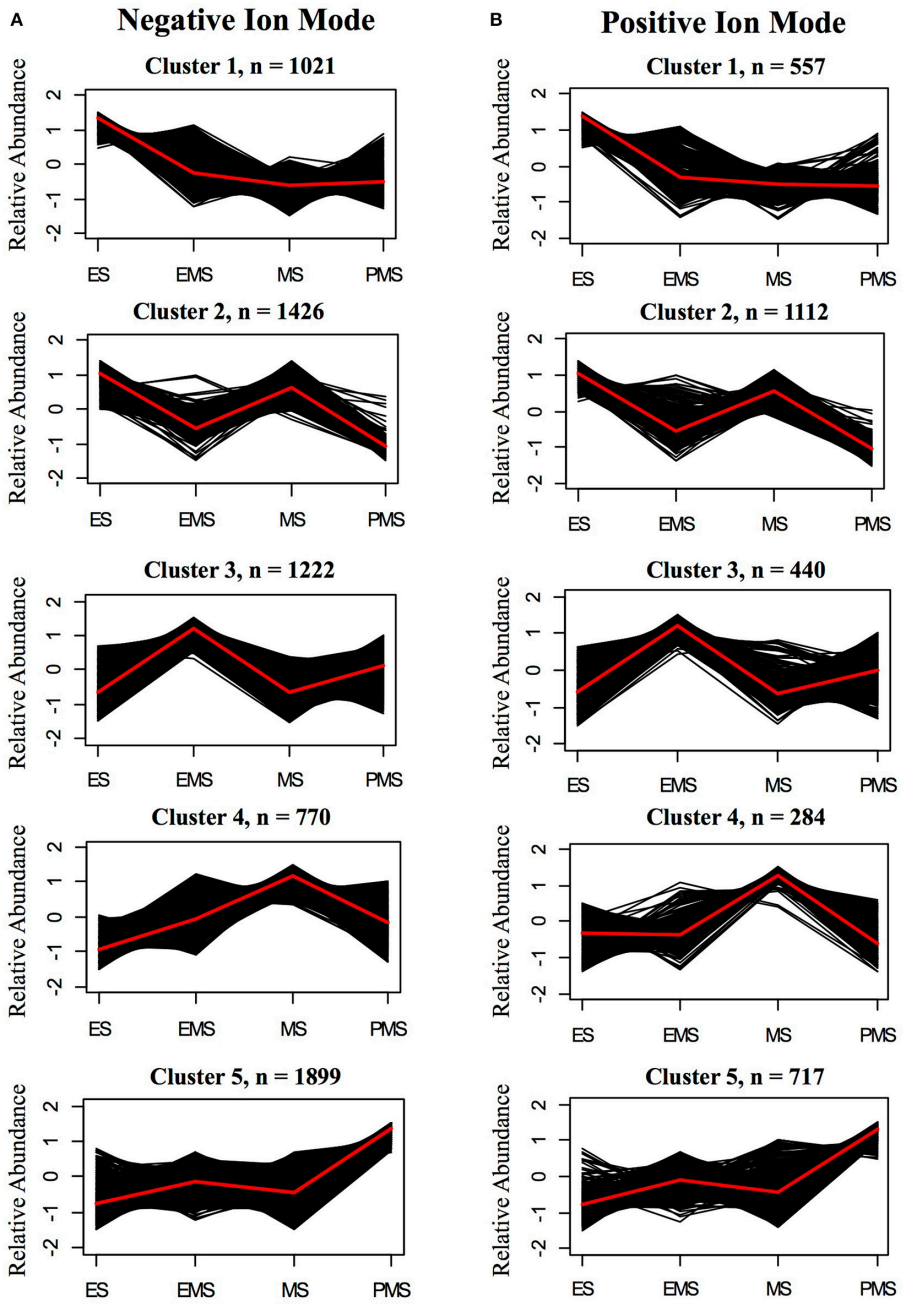
K-means clusters of metabolites that change significantly during stem internode maturation in **(A)** negative ion mode **(B)** positive ion mode. The sampled stages are as follows: elongating stem (ES), early mature stem (EMS), mature stem (MS), and post mature stem (PMS). Metabolite ions that vary with a *q*-value < 0.01 by ANOVA were used for clustering analysis. The number of clusters was determined by silhouette plot. Metabolites that change in a similar pattern were grouped into five clusters. Relative abundance indicates the *z*-score which has the following equation: *z*-score = (*x* − μ)/σ, in which *x* is the average abundance of each metabolite in a given stage and μ and σ are the mean and standard deviation, respectively, for the metabolite across all four stages. Black lines show the *z*-score of each metabolite ion in the cluster and the red line shows the average for the cluster. The total number of metabolite ions in each cluster is indicated by n.

Most metabolite ions varied significantly among organs and across stem development. As expected, principal component analysis (PCA) showed clear metabolic differences between stem metabolite profiles and those of roots and leaves (Figures 8A,B). PCA of just the ions from stem samples mostly separated the different stages from each other, consistent with differences in metabolite profiles during stem development (Figures 8C,D). Indeed, 6,338 negative metabolite ions and 3,110 positive metabolite ions varied significantly across stem development (ANOVA, *q*-value < 0.01; Tables S10, S11). The ions verified with authentic standards, *p*CA and apigen, varied significantly among organs though not across internode stages (Figure S7). K-means clustering of the metabolite dataset z-scores arranged in developmental order show that the metabolites are well-described by five clusters, with similar cluster patterns in the positive and negative ionization modes (Figure 9). The five clusters correspond to metabolites that predominate in each of the four developmental stages (1, 3, 4, and 5, respectively) and cluster 2, which contains ions that are most abundant in both the ES and EMS samples. Table 4 lists the number of ions identified in each ionization mode for each cluster and some tentatively identified metabolites, with additional identifications listed in Tables S10, S11. For example, cluster 1 metabolites were most abundant in ES. This cluster in negative ion mode contained 1,021 metabolites including coniferyl aldehyde. Cluster 1 in positive ion mode contained 667 metabolites including methyl cinnamate.

**Table 4:** Metabolites enriched at different stages of rice stem internode development.

**K-means cluster^a^**	**Enriched sample(s)^b^**	**ESI**−^**c**^		**ESI**+^**c**^	
		**Metabolite ions^d^**	**Representative metabolite^e^**	**Metabolite ion^d^**	**Representatives metabolite^e^**
Cluster 1	Elongating stem (ES)	1,021	coniferyl aldehyde	667	Methyl cinnamate
Cluster 2	Elongating stem (ES) and mature stem (MS)	1,426	NCI	1,112	6,7-dihydroxycoumarin, phenylacetaldehyde
Cluster 3	Early mature stem (EMS)	1,222	NCI	440	Demethoxycurcumin, xanthotoxol
Cluster 4	Mature stem (MS)	770	NCI	284	Benzaldehyde, olivetol
Cluster 5	Post mature stem (PMS)	1,899	isoscoparin 2-(6-(E)-p-coumaroylglucoside), isoscoparin 2-(6-(E)-ferulylglucoside)	717	Tricin 7-glucoside

a *Clusters of metabolites were determined based on abundance within the dataset*.

b *Sample or samples in which metabolites in the cluster are most abundant*.

c *Charge of the electrospray used to ionize metabolites prior to mass spectrometry. ESI−, negative electrospray ionization mode; ESI+, positive electrospray ionization mode*.

d *The number of metabolite ions in the cluster. The retention time and mass-to-charge ratio of detected ions can be found in Tables S10, S11*.

e *Examples of tentatively annotated metabolites. Other annotated metabolites can be found in Tables S10, S11. NCI indicates no confident identifications*.

By examining the Plant Metabolic Network, we also found some enzymes related to the identified metabolites present in the rice ES internode II according to our proteome data. For example, C4H (LOC_Os05g25640), which synthesizes *p*CA from cinnamic acid and CCR (LOC_Os08g34280), which synthesizes coniferyl aldehyde from feruloyl-CoA are both present in the ES internode II and their direct products were detected. Two putative tricin synthases (LOC_Os08g38900, LOC_Os08g38910) that synthesize tricin from tricetin, which is synthesized from apigenin, were also present. Their presence together with the presence of apigenin and tricin 7-glucoside, a tricin derivative, suggests the tricin synthesis pathway is active.

## Discussion

This study contributes novel grass stem proteomics, phosphoproteomics, and metabolite datasets toward understanding grass stem development and functions, specifically for the elongating rice internode. The basipetal developmental of the grass internode renders it an excellent system for gaining molecular understanding of secondary development in monocots. In a single internode, different developmental stages are available simultaneously under highly similar environmental conditions. This study complements recent transcriptome studies (Bosch et al., 2011; Hirano et al., 2013; Shen et al., 2013; Zhang et al., 2014; Martin et al., 2016), a lower-coverage proteome study on rice internode II at the milky stage (Yang et al., 2006), and cell wall proteome studies on sugarcane culms and *Brachypodium* stem internodes (Douché et al., 2013; Calderan-Rodrigues et al., 2016).

### Cell wall changes associated with stem development

The cell wall measurements of rice elongating internode II segments are mostly consistent with similar measurements on the 10th internode of maize (Zhang et al., 2014). The patterns of lignin, cellulose and xylose accumulation are similar to those measured by methylation-based linkage analysis during maize internode elongation. However, MLG and TFA-released glucose showed an initial increase and then decrease in the rice internode in contrast to the monotonic decrease of MLG in the maize internode (Figure 3). MLG accumulation in maize and rice may be different, or alternatively, the short length of the segments S1 to S4 in this study may have permitted us to detect variation in young cell walls, missed in the uniform 1 cm segments of Zhang et al. The general consistency of rice and maize internode cell wall profiles during development suggests that the rice proteomics data can be used to gain insight into cell wall development of other grasses.

We extended the previous internode cell wall profiling with measurements of hydroxycinnamoyl esters, which play an important role in cross-linking cell wall polymers in grasses (Buanafina, 2009). Both *p*CA and FA increased from young to old segments but showed different patterns of change, with *p*CA amounts continuing to increase through development but FA remaining constant in older segments. When integrated with gene expression or proteomics data of these segments, the difference in *p*CA and FA accumulation may facilitate identification of the transferases and other proteins that function in their incorporation, a topic of considerable recent interest (Molinari et al., 2013; Buanafina et al., 2016; Chateigner-Boutin et al., 2016; Lin et al., 2016).

### Cell wall synthesis, remodeling, and protein phosphorylation are important during stem elongation

This study applied different proteomics methods to unveil the protein catalog in the elongating rice internode II and provided technical guidance for future proteomics of internodes. Within the whole elongating rice internode II actively undergoing cell wall alterations, we detected 2,547 proteins, including several known or implicated in cell wall synthesis (Tables 2, 3, and Figure 6). These observations reinforce the importance of the identified proteins. As Experiment 2 used only minimal fractionation, these proteins are likely to be the most abundant representatives of their corresponding families. We detected proteins corresponding to almost the entire monolignol biosynthesis pathway (Table 2) (Humphreys and Chapple, 2002), including one member in the PAL, C4H, C3H, CCoAOMT, and CCR families, and two members in the CAD, HCT and 4CL families. The one or two enzymes detected at each step of phenylpropanoid pathway could be the major enzymes that catalyze monolignol biosynthesis in rice stem. We also detected 5 peroxidases that are good candidates for acting in monolignol polymerization. One of the identified GT2s is CESA1, a likely primary cell wall cellulose synthase in rice (Table 3) (Wang L. et al., 2010). We also detected OsIRX10, a GT47 involved in stem xylan synthesis. Mutants of this gene exhibit shorter stems, thinner secondary cell walls, and reduced cell wall xylose content (Chen et al., 2013). Among the six identified ATs, AT10 and AT4 have been previously reported to play a role in rice cell wall synthesis. AT10 incorporates *p*CA into arabinoxylan (Bartley et al., 2013), and OsAT4 functions as a *p*-Coumaroyl-CoA:monolignol transferase involved in lignin synthesis (Withers et al., 2012; Petrik et al., 2014). Functional data are also available for *Brachypodium* orthologs of rice OsAT3 and OsAT1 (Petrik et al., 2014; Buanafina et al., 2016). OsAT2 and OsAT9 have not been characterized but are candidate feruloyl transferases based on expression patterns and a phylogenetic study in *Brachypodium* (Molinari et al., 2013). The correlation between AT9 and FA abundance in rice above-ground tissues also support its role as a feruloyl transferase (Lin et al., 2016).

Many GHs we identified in the internode proteomics dataset are from families that may function in cell wall remodeling and defense responses (Figure 6) (Minic, 2008). GH3 and GH51 enzymes may remodel arabinoxylans. GH3s from barley seedlings and Arabidopsis stems have arabinofuranosidase or xylosidase activity (Lee et al., 2003; Minic et al., 2004). Two GH51s from barley have arabinofuranosidase activity on arabinoxylan (Ferre et al., 2000; Lee et al., 2001). These enzymes might be partially responsible for the decrease in arabinose:xylose ratio across internode development (Table S1). Other families may function on minor polysaccharides in grass cell walls or pathogen cell walls. For example, the GH27 proteins we detect may have galactosidase activity (Kim et al., 2002) and decrease galactose from young to old stem segments (Figure 3), though we cannot rule out that the decreases are caused simply by dilution due to addition of other components to the wall. Some rice GH16 enzymes exhibit xyloglucan endotransglucosylase and xyloglucan endohydrolase activities (Hara et al., 2014). GH17s have been found to be endo-1,3-β-glucosidases that degrade pathogen cell walls, functioning in defense (Minic, 2008). Also notable, GH38s have mannosidase activity in bacteria, but unknown functions in plants (Suits et al., 2010).

We identified several protein kinases and novel phosphopeptides in the elongating internode (Tables S7 and S9), consistent with a role for protein phosphorylation in controlling stem development. Currently, protein kinases with cell wall phenotypes or that regulate cell wall genes are mainly in three kinase groups. These include the LRR (Leucine-rich repeat) receptor like kinases, such as THE1 and FEI1 from the Tyr-kinase-like (TKL) group; MAPK6, which is in the CMGC (CDK, MAPK, GSK3, and CLK) group; and CDK28, from the CAMK (calcium/calmodulin-dependent protein kinase) group (Morse et al., 2009; Oh et al., 2011; Matschi et al., 2013). More than half of kinases identified in this study are from these three groups. One of the kinases from the TKL group that we identified is a putative ortholog of the BRASSINOSTEROID INSENSITIVE 1-ASSOCIATED RECEPTOR KINASE 1 (BAK1, LOC_Os02g09359). In Arabidopsis, BAK1 can form a heterodimer with BRASSINOSTEROID INSENSITIVE 1 (BRI1) in the presence of brassinosteriod to regulate plant development including inflorescence stem growth and secondary cell wall formation (Nam and Li, 2002).

Non-RD kinases, i.e., those that lack a conserved RD amino acid sequence in kinase subdomain IV, are predicted to function in pathogen recognition, while RD kinases are likely to be involved in other biological processes (Dardick and Ronald, 2006; Dardick et al., 2007). The under representation of non-RD kinases in the elongating stem is consistent with pathogen detection not being a major function in the stem under our growth conditions, though could also be due to low expression of non-RD kinases relative to RD-kinases, leading to lack of consistent detection.

Many kinases processed through the secretory pathway are associated with plasma membrane and cell walls and may have a role in both pathogen response and cell expansion during development (Park et al., 2001; Hematy et al., 2007; Kohorn and Kohorn, 2012). Six of the kinases detected in the internode are likely to be processed through the secretory system. Furthermore, we identified a novel phosphopeptide from a membrane-associated LRR-III family receptor-like kinase. Autophosphorylation of LRR receptor like kinases is important for activation of plant growth regulation, disease resistance, and stress response signaling pathways (Tor et al., 2009; Mitra et al., 2015).

Though many known or hypothesized cell wall-related proteins were identified, others were absent from the dataset, especially transcription factors from the MYB and NAC families that control cell wall synthesis. Coverage in future experiments can be improved by conducting further fractionation to reduce the complexity of protein samples, such as subcellular fractionation; additional LC separation (as used in Experiment 3); or binding to a random library of ligands to enhance low abundance proteins in a sample, i.e., combinatorial peptide ligand libraries (Wang H. et al., 2010; Li et al., 2013; Righetti and Boschetti, 2016).

### Secondary metabolites vary during stem maturation

During stem development, we observed that many metabolites change significantly. The vast diversity of secondary metabolites in plants evolved as protection against pathogens, insects, and animals (Dixon, 2001; Gershenzon and Dudareva, 2007; War et al., 2012), including functioning indirectly by alerting predatory insects to herbivory and other chemical ecological effects (Schuman and Baldwin, 2016). Abundance of some rice pathogen-repelling metabolites varies across development. For example, diterpenoid phytoalexins, which repel rice blast fungus, are less abundant in young rice leaves relative to old leaves (Kodama et al., 1988). Susceptibility to insects like stemborers also differs during rice development (Viajante and Heinrichs, 1987; Bandong and Litsinger, 2005). For example, rice is susceptible to yellow stemborer at flowering but not at pre-booting or after panicle emergence (Viajante and Heinrichs, 1987).

Correlation between rice metabolite abundance and pathogen and pest resistance across development might indicate a role for metabolites in resistance (Gardner, 1977; War et al., 2012). For example, 6,7-dihydroxycoumarin (esculetin) and phenylacetaldehyde were more abundant in the elongation and early mature stems (ES and EMS) but less abundant in the mature and post mature stems (MS and PMS). Esculetin has fungi-toxicity and may be involved in defense (Gomez-Vasquez et al., 2004). On the other hand, tricin 7-glucoside, a phagostimulant that triggers feeding in insects like the small brown planthopper (Adjei-Afriyie et al., 2000; Bouaziz et al., 2001), remained relatively low until the post-mature stage, when grains are fully mature.

### Summary

This study catalogs cell wall changes, protein abundance, and secondary metabolite profiles of rice elongating internodes. The novel and relatively deep proteomics data reveal the presence of metabolic enzymes, including those for cell wall synthesis and remodeling, in agreement with the changes we observe in cell wall components. In addition, the metabolite profile changes associated with stem maturation could indicate potential defensive roles of secondary metabolites during plant development. The availability of the protein catalog and metabolite profiles provides a crucial tool for understanding fundamental molecular processes of grass stem development, over inferences from transcriptomics alone. These data will facilitate identification of protein targets for optimizing stem development and cell wall composition of grasses to improve their agronomic properties and downstream uses.

## Materials and methods

### Rice growth conditions and sample harvest

Following an adaptation of a published protocol (Moulton et al., 2008), *Oryza sativa* ssp. *japonica* cv. *Kitaake* seedlings were grown in a mixture of Turface Athletics medium:vermiculite (1:1) in a greenhouse at 28–33°C during the day and 21–25°C during the night. Natural day lengths less than 13 h were supplemented with artificial lighting. Beginning 2 weeks after germination, plants were fertilized three times per week with JACKS PROFESSIONAL LX 15-5-15 4CA2MG fertilizer. Approximately 3–4 weeks after sowing, plants were fertilized with 100 mL per pot of 0.08 M Fe-EDTA (pH 5.5) (Steiner and Van Winden, 1970).

Plant samples were collected at development stages defined by the “Biologische Bundesanstalt, Bundessortenamt und CHemische Industrie” (BBCH) identification keys of rice (Lancashire et al., 1991). Stem internode II (the second internode from the top) samples at booting stage (BBCH stage 45) were harvested as the elongating stem (ES) material for cell wall, proteomics, and soluble metabolite profiling. At this stage, the top of the panicle is 0–1 cm beneath the top of the flag leaf sheath. Internode II samples were cut immediately above node II, where the leaf sheath and axillary bud attach to the stem, and at the dark green ring below node I. Axillary buds were carefully removed. For metabolite profiling, additional internode II samples at the flowering stage (BBCH stage 65), grain filling stage (BBCH stage 75) and mature seed stage (BBCH stage 92) were harvested as early mature stem (EMS), mature stem (MS), and post mature stem (PMS), respectively. The flag leaf blade and all roots of plants in the grain filling stage (BBCH stage 75) were harvested as mature leaf and root samples for metabolite profiling. Root samples were washed with de-ionized water for 5 min. All samples for cell wall assays, proteomics, and metabolite profiling were immediately frozen in liquid nitrogen and stored at −80°C.

### Microscopy

We performed phloroglucinol staining on cross-sections of the internode II of ES as previously described (Liljegren, 2010). Internode sections were stained with 50 μL phloroglucinol solution for 2 min on slides and examined immediately after adding 50 μL 50% (v/v) HCl. The sections were observed with a Leitz Dialux 20 microscope with a Leitz Wezlar 10x lens and imaged with an Olympus DP71 camera on automatic exposure mode controlled by DP controller software (ver 3.1.1.267).

### Cell wall analysis

For cell wall assays, 20 internode II samples at ES with an average length of 10 cm were unevenly divided into segments. Identical segments from 5 to 6 different internodes were pooled into biological replicates, with 3 to 4 biological replicates collected, depending on the assay. The eight uneven segments from the base of internode II were as follows: S1–S4, the first four successive 5 mm segments; S5–S7, the next three 10 mm segments; and S8, the remainder of the internode. The uneven sampling strategy was designed based on the phloroglucinol staining data, which revealed rapid changes in lignin near the base of the internode, consistent with previously observed rapidly changing cell wall content in the basal part of the maize internode 10 (Zhang et al., 2014). The S1 segment is located right above node II. Previous work has shown that the intercalary meristem is about 2 mm above the node (Kende et al., 1998). The tissue below the meristem formed prior to internode elongation and therefore is relatively mature.

For all cell wall assays, fresh-frozen internode II segments (S1–S8) were ground, made into alcohol insoluble residue (AIR), destarched, and assayed for cell wall composition as described previously (Bartley et al., 2013). Briefly, hemicellulosic and pectic monosaccharides were released with 2 M TFA at 120°C for 2 h and measured by high performance ion exchange chromatography. The TFA-insoluble pellet was used for cellulose measurements via an anthrone assay. Lignin was measured by solubilization with 25% acetyl bromide in glacial acetic acid followed by absorbance measurements at 280 nm with a micro-plate reader (Bartley et al., 2013). HCAs were released by incubation with 2 M NaOH at 25°C for 24 h and measured by a high performance liquid chromatography with a UV detector (Bartley et al., 2013). MLG was measured with a lichenase-based kit (Megazyme, K-BGLU) as described previously (Vega-Sanchez et al., 2012). The mass of all cell wall components was calculated by summing all components, but excludes the lichenase-based MLG measurement, since MLG also contributes to the monomeric glucose released by TFA hydrolysis. Three biological replicates were used for all experiments expect the lignin assay, which used four biological replicates. The R general package was used for data analysis.

### Protein extraction and peptide preparation

For Experiment 1 and Experiment 2, total proteins were extracted from a pool of six developmentally and morphologically matched ES internode II samples with a phenol-based method previously described (Saravanan and Rose, 2004; Lee et al., 2010). Briefly, plant material was ground with a mortar and pestle with liquid nitrogen and re-suspended with extraction buffer consisting of 0.1 M Tris-HCl (pH 8.0), 10 mM EDTA, 0.9 M sucrose, 0.1% DTT, 1% protease inhibitor cocktail (Sigma P9599). Proteins were extracted with an equal volume of Tris buffered phenol (pH 8.8) for 30 min at 4°C, and then precipitated and washed three times with 0.1 M ammonium acetate. The protein pellet was dried at room temperature and solubilized with 7 M urea, 50 mM Tris-HCl, pH 8.0. Protein samples were quantified with a BioRad Protein Assay Kit using BSA as a standard. For peptide preparation, the protein samples were divided into 150 μg aliquots and loaded on three lanes of a 12% (w/v) polyacrylamide gel with a 5% (w/v) stacking gel. Sodium dodecyl sulfate polyacrylamide gel electrophoresis was conducted on a BioRAD gel Cryterion™ apparatus at an initial voltage of 120 V for 10 min followed by 100 V constant voltage in 1X Tris-Glycine running buffer. The gel was stained with Coomassie brilliant blue and cut into 5 horizontal slices to separate the proteins by apparent molecular mass. Each gel slice was handled separately and diced into 1 mm cubes and incubated in destaining solution (200 mM ammonium bicarbonate and 40% acetonitrile) for 30 min twice at 37°C. The five destained gel slices were dried and digested with proteomics-grade trypsin (Sigma, T6567). The five resulting peptide samples were equally split by volume for Experiment 1 and Experiment 2 prior to lyophilization.

For Experiment 3, ES ground internode II tissue from 15 internodes was divided into four 15 mL centrifuge tubes (1.5 g/tube) containing 15% (w/w) Polyvinylpolypyrrolidone (PVPP) representing four technical replicates. To each tube, 10 mL of 0.1 M ammonium acetate in MeOH with 2% β-mercaptoethanol was added and incubated at −20°C for 2 h. Samples were centrifuged at 4,500 × g at 4°C for 10 min and the supernatant was discarded. The wash step was repeated 3 times, with the incubation step included between washes. After removing the supernatant from the final wash, pellets were dried using a Turbo Vap (Biotage, Charlotte, NC) under a stream of nitrogen for 10 min. Four mL of a protein solubilization solution containing 7 M urea, 2 M thiourea, 4% 3-[(3-cholamidopropyl) dimethylammonio]-1-propanesulfonic, 10 mM tris(2-carboxyethyl)phosphine in 100 mM NH_4_HCO_3_ was added and the samples were stored overnight at 4°C, then sonicated for 1 min in a sonication bath (Branson, Danbury, CT). Following sonication, samples were incubated for 30 min at 60°C with shaking at 1,400 rpm, then briefly sonicated, vortexed, and centrifuged at 4,500 × g, 4°C for 10 min. Prior to digestion, a Coomassie assay was performed on the supernatants containing the proteins (Bradford, 1976).

For trypsin digestion (with alkylation using chloroacetemide, 5 mM final concentration), the supernatants from each technical replicate were transferred to 50 mL tubes and diluted 10-fold with 50 mM NH_4_HCO_3_ and 100 μL of 1 M CaCl_2_ was added. In a 1:500 (w/w) trypsin:protein ratio, 10 mg/mL sequencing grade, modified trypsin was added and the samples were incubated at 37°C for 3 hrs with gentle shaking. Digested proteins were desalted and washed using 100 mg solid-phase extraction strong cation exchange (SPE-SCX) columns (Discovery DSC-SCX, Sigma-Aldrich, St. Louis, MO) on a GX-274 Liquid Handler (Gilson, Middleton, WI). Samples were concentrated using a SpeedVac SC 250 Express (Thermo Scientific, Waltham, MA). A second SPE-SCX wash step was performed using a vacuum manifold (Visiprep, Sigma-Aldrich, St. Louis, MO). Samples were concentrated in a SpeedVac and quantified with a Bicinchoninic Acid Protein Assay (BCA) (Smith et al., 1985) prior to storage at −70°C.

For 4-plex iTRAQ™ labeling, 200 μg of peptides from each of the 4 technical replicates were dried in a SpeedVac and 30 μL of 1 M triethylammonium bicarbonate (TEAB) was added to the dried peptides (Abdi et al., 2006). Labeling was performed following the manufacturer's instructions (SCIEX, Framingham MA). To hydrolyze the samples, 300 μL of water was added prior to combining the labeled samples together. The combined samples were concentrated and washed using SPE C18. The eluted sample was concentrated and a BCA assay was performed.

To reduce masking of similar peptides during MS and increase depth, a high-pH reversed phase liquid chromatography separation (HPRPLC) was performed using an Agilent 1100 HPLC System (Agilent, Palo Alto, CA) equipped with a quaternary pump, degasser, diode array detector, peltier-cooled autosampler, and fraction collector (set to 4°C for all samples). The separation columns consisted of an XBridge C18 250 × 4.6 mm analytical column containing 5 μm particles and equipped with a 20 × 4.6 mm guard column (Waters, Milford, MA) (Wang et al., 2011). Ninety-six fractions were collected into microwells, with the first fraction collected after 15 min into the gradient. Fractions were combined in rows into 12 fractions, then concentrated using a SpeedVac.

### Mass spectrometry and peptide identification for experiment 1

The LC-LTQ Orbitrap XL (Thermo Fisher Scientific Inc., Waltham, MA, USA) was run at the University of Missouri, Proteomics Center. Samples were resuspended in 21 μL aqueous solution with 5% acetonitrile and 1% formic acid to a final concentration of 1 to 1.8 μg/μL. Samples were vortexed, centrifuged, and transferred to autosampler vials. The vials were placed in a thermostatted (7°C) autosampler in the Proxeon Easy nLC system. A full loop injection (18 μL) of each sample was loaded onto a C8 trap column (Pepmap100 C8 trap column Dionex/Thermo Scientific). Bound peptides were eluted from this trap column onto a 25 cm × 150 μm inner diameter pulled-needle analytical column packed with HxSIL C18 reversed phase resin (5 μm particle size, 100 Å pore size, Hamilton Co). Peptides were separated and eluted from the analytical column with a step gradient of solvent A (0.1% formic acid in water) and solvent B (99.9% acetonitrile, 0.1% formic acid) at 400 nL/min at room temperature. Mobile phase conditions were 5% B, ramping to 20% B from 2 min to 20 min, then ramping to 30% B at 57 min, then ramping to 90% B at 62 min, hold at 90% B from 62 to 84 min, back to 5% at 85 min, and hold at 5% B until 90 min.

For the LC-LTQ Obitrap XL spectrometer, nanoElectrospray ionization (positive-ion) was used. FTMS data were collected at a resolution of 30,000, 1 microscan, 300–1,800 m/z, profile and a cycle of approximately 3 s. The 9-most-abundant peptides were selected for ion-trap collision-induced dissociation MS/MS (2 m/z mass window, 35% normalized collision energy, centroid). Dynamic exclusion was enabled with the following parameters: repeat count 1, repeat duration 30 s, exclusion list 500, and exclusion duration 180 s.

The results of LC-LTQ Obitrap XL were analyzed by Proteome Discoverer (version 1.4, Thermo Scientific) and Scaffold (Searle, 2010). The rice protein sequence FASTA file downloaded from the Rice Genome Annotation Project (Release 7, http://rice.plantbiology.msu.edu/index.shtml) (Ouyang et al., 2007) was imported and indexed for searching in Proteome Discoverer. A SEQUEST HT search (Eng et al., 1994) and filtering was performed with the following parameters: tryptic peptides with a maximum mis-cleavage of 2, mass range 350–5,000 Da, peptide mass tolerance 25 ppm, fragment mass tolerance 0.8 Da, carbamidomethyl Cys and Met oxidation as variable mods. Data were then exported to Scaffold and filtered with a peptide mass tolerance of 10 ppm, peptide confidence >95%, peptide per protein ≥2, protein confidence >99%, and peptide FDR <5%. Search results were exported to Scaffold and the results from different gel-slices were merged (Searle, 2010).

### Mass spectrometry and peptide identification for experiment 2

For the proteomics with the LC-HDMS^E^ (Bond et al., 2013), peptide samples were resuspended in 3% acetonitrile with 0.1% TFA to a final peptide concentration of about 3–30 ng/μL. Variable injection volumes were used for each sample to obtain peptide loads of 100 ng. An exogenous protein standard, trypsin-digested yeast alcohol dehydrogenase (ADH1_YEAST), was added to a final concentration of 1 fmol/μL. A Symmetry C18 column (180 μm × 20 mm) was used as the trap column and a C18 HSS T3 column (75 μm × 150 mm) was used as the analytical column (Waters Corporation, Milford, MA). Nanoflow UPLC was performed at a flow rate of 500 nL/min. The LC gradient consisted of solvent A (0.1% formic acid in water) and solvent B (99.9% acetonitrile, 0.1% formic acid) starting at 5% B and ramping to 35% B over 90 min, then ramping to 50% B at 95 min, then ramping to 85% B at 97 min, back to 5% at 107 min and re-equilibrated until 120 min. Each sample was run in triplicate for statistical evaluation of technical reproducibility.

A hybrid ion mobility quadrupole time-of-flight mass spectrometer, SYNAPT G2-S*i* HDMS (Waters, Wilmslow, UK) was used to identify and quantify the relative abundances of the tryptic peptides. LC-MS/MS experiments were acquired using ion mobility assisted data-independent analysis acquisition mode similar to that previously described (Distler et al., 2014). Data were collected at 25,000 resolution, 50–2,000 m/z, 0.8 s/scan. An ion mobility-dependent collision energy ramp (UDMS^E^) method was used to generate the data-independent fragment ion spectra (Distler et al., 2016). Briefly, a linear collision energy ramp from 17 to 60 V was applied along the 20–200 ion mobility drift bin scale (total of 200 drift bins) using similar ion mobility tune settings.

The results of the LC-HDMS^E^ experiment were analyzed with Progenesis™ QIP v2.0 for proteomics (Waters, Wilmslow, UK). Each gel fraction of the three technical replicates was imported and chromatographically aligned. The peptides were identified by a previously described ion accounting algorithm (Li et al., 2009) with the rice protein sequence FASTA file mentioned previously. The search parameters were: tryptic peptides with a maximum mis-cleavage of 1, peptide mass tolerance 20 ppm, max protein mass 250 kDa, default protein modification (carbamidomethyl Cys and Met oxidation as variable mods), fragments per peptide ≥3, fragments per protein ≥7, FDR <4%. Individual gel fractions were combined for each technical replicate to create a combined protein list. Proteins identified by at least two unique peptides are reported. Proteins were quantified by the average of the five most abundant peptides or of all peptides if the number of peptides was less than five (Silva et al., 2006). The relative amounts of proteins with no less than two unique peptides were calculated based on the response factors (ion intensity per fmol) of the alcohol dehydrogenase standard for each run.

### Mass spectrometry and peptide identification for experiment 3

Peptides were separated for MS analysis using a Waters nano-Acquity UPLC system (Waters, Milford MA) configured for on-line trapping of a 5 μL injection (0.1 μg/μL peptide concentration) at 3 μL/min with reverse direction elution onto the analytical column at 300 nL/min. Separation occurred by way of in-house packed columns using Jupiter C18 media (Phenomenex, Torrence, CA), 5 μm particle size for the trapping column (100 μm × 4 cm length) and 3 μm particle size for the analytical column (75 μm i.d. × 70 cm length) coupled to HF etched fused silica tips (Kelly et al., 2006). Mobile phases consisted of (A) 0.1% formic acid in water and (B) 0.1% formic acid in acetonitrile with the following gradient profile (min, %B): 0, 1; 2, 8; 20, 12; 75, 30; 97, 45; 100, 95; 110, 95; 115, 1; 150, 1.

Tandem mass spectra (MS/MS) were generated for each fraction using high-energy collision dissociation (HCD) coupled to a Q Exactive Plus mass spectrometer (Thermo Scientific, San Jose, CA) outfitted with a house-made nano-electrospray ionization interface. The ion transfer tube temperature and spray voltage were 325°C and 2.2 kV, respectively. Data were collected for 100 min following a 15 min delay from sample injection. FTMS spectra were acquired from 400 to 2,000 m/z at a resolution of 35 k (Automatic gain control target 3 × 10^6^) and while the top 10 FT-HCD-MS/MS spectra were acquired in data dependent mode with an isolation window of 2.0 m/z and at a resolution of 17.5 k (Automatic gain control target 1 × 10^5^) using a normalized collision energy of 30 and a 30 s exclusion time.

Spectra were converted to .dta files using Bioworks Cluster 3.2 (Thermo Fisher Scientific, Cambridge, MA) and amino acid sequences assigned to tandem mass spectra using the MS-GF+ search algorithm (Kim and Pevzner, 2014) against the IRGSP-1.0_protein.fasta (2016) database for *O. sativa*. Search parameters consisted of a 10 ppm tolerance for precursor ion masses, and a ± 0.5 Da window on fragmentation masses, allowed dynamic modifications included oxidation of methionine (15.9949 Da), and static modifications included IAA alkylation of cysteine (57.0215 Da), and iTRAQ modification of lysine and N termini (+144.1021). Peptides were filtered to achieve a false discovery rate of ≤1% using spectral probability values generated by the search algorithm. A protein was considered positively observed if two or more unique peptides were measured for it across all iTRAQ labeled replicates. The ID of identified proteins was converted to Rice Genome Annotation Project ID with the converter at OryzaExpress: http://bioinf.mind.meiji.ac.jp/OryzaExpress/ID_converter.php.

### Sample preparation and mass spectrometry for phosphoproteomics

For phosphoproteomic analysis, a separate biological sample of six developmentally matched internode II of ES were ground in liquid nitrogen, and a total of 500 μg of total proteins were separated via SDS-PAGE, extracted and digested with the same methods as Experiment 1 and 2 but with the addition of 1% Phosphatase Inhibitors cocktails 1 and 2 (Sigma P2750 and Sigma P5726) during trypsin digestion. Tryptic digested peptides were enriched for phosphopeptides using a Pierce™ TiO2 Phosphopeptide Enrichment and Clean-up Kit (Thermo Fisher Scientific, 88,301 and 88,303) (Larsen et al., 2005). The eluted phosphopeptides were acidified with 2.5% TFA to pH 2.0 to 2.5, cleaned with graphite column, lyophilized, and stored at −80°C.

Phosphoprotein data were generated under contract by Pepproanalytics, LLC. Lyophilized tryptic phosphopeptides were resuspended in 25 μL of 0.1% formic acid and 10 μL was loaded. Peptides were desalted using C8 Captraps (Bruker-Michrom Bioresources, Auburn, CA). Chromatographic separations were achieved using a 55 min linear acetonitrile gradient (5–35% acetonitrile, 0.1% formic acid) with a column packed with “Magic C18” (200 Å, 5 μm bead, Bruker- Michrom Bioresources) stationary phase. The phosphoproteomics was conducted by LTQ Orbitrap XL with electron-transfer dissociation (Thermo Fisher Scientific, San Jose, USA). Survey scans (MS1) used the following settings: analyzer: FTMS; mass range: normal; resolution: 60,000; scan type: positive; data type: centroid; scan ranges: 200–2,000 m/z. The top 7 masses from the survey scans were selected for data dependent acquisitions. Data dependent scan settings were the following: analyzer: ion trap; mass range: normal; scan rate: normal; data type: centroid. Dynamic exclusion, charge state screening and monoisotopic precursor selection were enabled. Unassigned charge states and masses with a charge state of one were not analyzed. The “data dependent decision tree” option was enabled as previously described (Swaney et al., 2008), allowing for fragmentation of peptides using collision induced dissociation or electron transfer dissociation.

The phosphoprotoemics results were analyzed with Proteome Discoverer (version 1.3, Thermo Scientific). Acquired spectra were searched against the rice protein sequence FASTA file concatenated with a randomized decoy database. Proteome Discoverer search parameters included a mass range of 200–5,000 Da, positive mode polarity, signal-to-noise ratio of 3, and a minimum peak count of one. SEQUEST search parameters were static modification of cysteine-carboxyamidomethylation, dynamic/variable modifications of methionine-oxidation, and phosphorylation of Ser, Thr, and Tyr residues. Other search parameters included two mis-cleavages and precursor and fragment ion tolerances of 1.0 Da and 1,000 ppm, respectively. The peptide filter of “charge state vs. Xcorr” was enabled. Additional filters included peptide mass deviation of 50 ppm, maximum ΔCn of 0.01, and at least one peptide per protein. Phosphopeptides that passed these filters were further analyzed using the phosphoRS algorithm of Proteome Discoverer (Taus et al., 2011). The FDR was fixed at 4%.

### Proteomics data availability and analysis

The mass spectrometry proteomics data generated by Experiment 1 and Experiment 2 were deposited to the ProteomeXchange Consortium (http://proteomecentral.proteomexchange.org) via the PRIDE partner repository (Vizcaino et al., 2013) with the dataset identifier PXD003676. Experiment 3 data were deposited to the ProteomeXchange Consortium with the dataset identifier PXD006536.

The proteins common between Experiment 2 and Experiment 3 with at least 2 unique peptides were used for GO analysis, KEGG pathway mapping, phylogenomic database searches and cell wall proteomics database searches. GO-enrichment analysis was conducted at agriGO (http://bioinfo.cau.edu.cn/agriGO/analysis.php, v 1.2). For KEGG pathway searching, we identified KO terms of all possible proteins identified with an online KO analysis tool at the Rice Oligonucleotide Array Database (http://www.ricearray.org/analysis/overview.shtml). The identified KO terms were used for KO pathway searching and mapping with KEGG mapper (http://www.genome.jp/kegg/mapper.html). We searched all possible protein models of specific rice GT and GH families and of all transcription factor and kinases families in the rice phylogenomic database (http://ricephylogenomics.ucdavis.edu/description.shtml). We also searched WallProtDB to find previously identified extracellular proteins present in the dataset reported herein (http://www.polebio.lrsv.ups-tlse.fr/WallProtDB/index.php).

### Metabolomics and metabolite identification

For metabolite profiling, all samples were pooled from two plants per biological replicate and three biological replicates were collected. Metabolites were extracted from frozen ground material with a hot methanol-based method (Vanholme et al., 2012). Frozen samples were homogenized by Genogrinder and extracted with 1 mL methanol at 70°C for 15 min. After 3 min centrifugation at 15,000 rpm, 300 μL of supernatant was transferred to a new tube and lyophilized for storage. Samples were dissolved with 400 μL of cyclohexane and water (1:1) before use.

For LC-MS/MS, parallel experiments were conducted in positive electrospray ionization (ESI+) and negative electrospray ionization (ESI-) mode with all three biological replicates. Each sample was run in triplicate to ensure technical reproducibility. A pooled sample was made of equal amount of each sample and run in quintuplicate. For each run, 2 μL was injected for metabolite profiling using liquid chromatography with an ACQUITY HSS C18 column (2.1 × 150 mm 1.7 μm Particle) coupled with a Waters SYNAPT G2-Si mass spectrometer. For UPLC, a gradient of two buffers was used: buffer A (99/1/0.1, water/acetonitrile/formic acid, pH 3.0, formic acid was not used for ESI- mode), buffer B (99/1/0.1 acetonitrile/water/ formic acid, pH 3.0); 95% A for 0.1 min decreased to 50 % A in 30 min (flow rate was 350 μL/min, column temperature was 40°C). For mass spectrometry, MS^E^ was used for the acquisition mode, in which collision energy cycles between a low-energy state and a high-energy state, yielding fragment patterns for a large fraction of the detected metabolite ions. MS conditions were as follows: mass range 50–1,200 Da, scan speed 0.1 s per scan, source temperature 120°C, desolvation temperature 500°C, desolvation gas 1,200 L/h, capillary 1.0 KV, cone voltage 20 V, source offset 80 V, cone gas 50 L/h, resolution 20,000 (full width at half maximum), high energy collision ramp 30–50 eV.

Peaks were aligned, picked, normalized, and identified using Progenesis QI for metabolomics analysis (v 2.1) with the settings stated below. Automatic peak picking was set to default sensitivity and the signal before 2 min was removed. Every sample was normalized to an automaticly determined reference sample based on all compounds. We applied ChemSpider to search against a Plant metabolic pathway database (PlantCyc) and MetaScope to search against a custom rice metabolite database extracted from the Human Metabolome Database. For both methods, the identification parameters were: precursor mass tolerance 5 ppm, theoretical fragmentation was performed, fragment mass tolerance 5 ppm. Mass similarity, isotope distribution similarity and fragmentation score were used to calculate the identification score for each peak (maximum 60). Fragmentation score was calculated based on the match with theoretical fragmentation (Wolf et al., 2010). Peaks with scores ≥50 were tentatively annotated with the highest score. The presences of *p*CA and apigenin were verified by running standards and a pool of stem samples.

Statistical analyses, including principal component analysis, analysis of variance (ANOVA), and k-means clustering were performed in R (v 3.12). For all analyses, we only used metabolite ions that were consistently present or absent in the technical replicates of all samples (with an average consistency score of at least 0.4 in all sample). *Consistency score* = $\left|\frac{s}{n}-0.5\right|$, where *s* is the number of observations in *n* technical replicates. For example, if a metabolite is present in 4 out of 5 technical replicates of the pool sample, it has a consistency score of 0.4. Technical replicates were averaged before analysis. For k-means clustering, we first performed k-means clustering with *k* = 2–20 and compared the silhouette plot and average silhouette width of the results. We used *k* = 5 for clustering since it gave high silhouette coefficients in most clusters and had fewer outliers. Sample ES-3 and PMS-1 were slightly out of stage when collected, and therefore excluded from the ANOVA and clustering analysis.

## Author contributions

LB, PT, and FL conceived of this study and designed the experiments. PT and FL prepared samples for proteome and metabolite profiling. BW performed LC-MS/MS and data processing for Experiment 2. AS and SC performed LC-MS/MS and data processing for Experiment 3. AL and HO performed LC-MS/MS for metabolite profiling. FL and KZ analyzed and interpreted the data; FL and LB drafted the manuscript. All authors revised the manuscript critically and approved the final version for publication.

### Conflict of interest statement

BW, AL, and HO are employed by Waters Corporation. The other authors declare that the research was conducted in the absence of any commercial or financial relationships that could be construed as a potential conflict of interest.

## Abbreviations

AIR: alcohol insoluble residue
ANOVA: analysis of variance
AT: acyltransferase
BBCH: “*Biologische Bundesanstalt, Bundessortenamt und CHemische Industrie*” development scales
*p*CA: *p*-coumarate
CAMK: calcium/calmodulin-dependent kinase group; CMGC, CDK, MAPK, GSK3 and CLK kinase group
EMS: early mature stem
ES: elongating stem
ESI: electrospray ionization
FA: ferulate
GH: glycosyl hydrolases
GO: gene ontology
GT: glycosyltransferase
HCA: hydroxycinnamic acid
HDMS^E^: high definition mass spectrometer with data-independent analysis
iTRAQ: isobaric tags for relative and absolute quantitation
KO: KEEG Ontology
LRR: leucine-rich repeat
MLG: mixed linkage glucan
MS: mature stem
MS/MS: tandem mass spectrometry
P3DB: plant protein phosphorylation database
PCA: principal component analysis
PMS: post mature stem
RD motif: conserved arginine motif
TFA: trifluoroacetic acid
TKL: Tyr-kinase-like kinase group.
