# Supplementary material for: Proteomics Coupled with Metabolite and Cell Wall Profiling Reveal Metabolic Processes of a Developing Rice Stem Internode
Source: Front Plant Sci. 2017 Jul 13;8:1134. doi: 10.3389/fpls.2017.01134 (PMC5507963; doi:10.3389/fpls.2017.01134)
Supplement: Supplementary file 12 [file Presentation1.pdf]

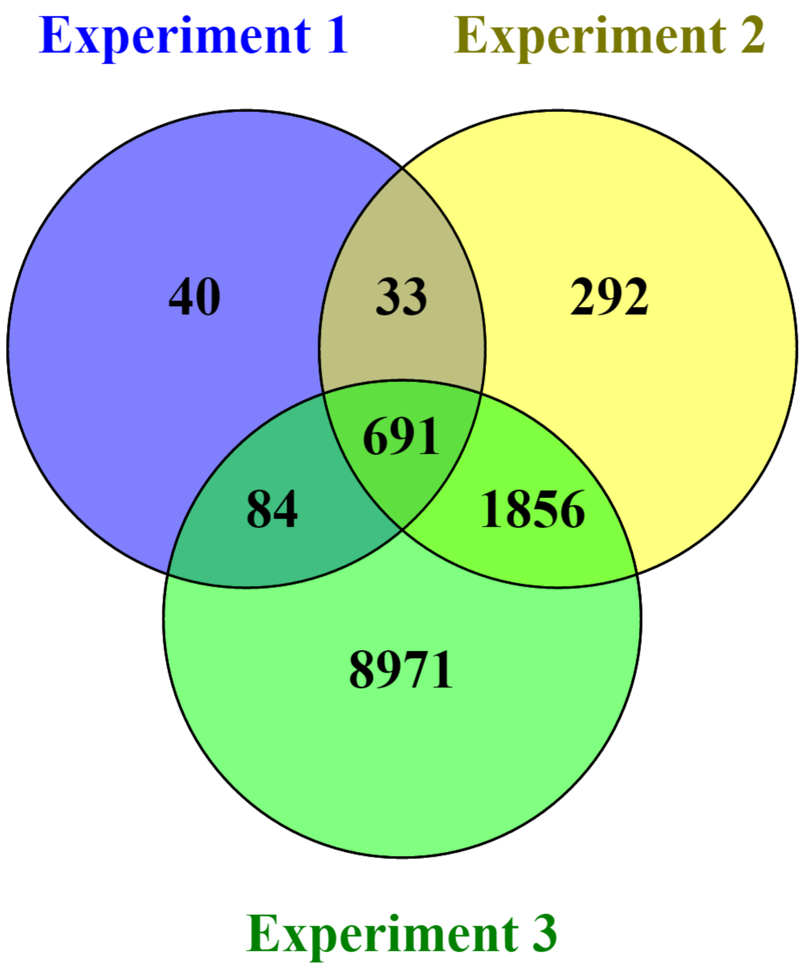

Supplemental Figure 1. Proteins identified by at least two unique peptides in three proteomics experiments on the whole internode II of rice elongating stem. Different instruments and analysis methods were used for each experiment as described in Table 1. Experiment 1 and Experiment 2 were conducted on identical peptide samples prepared from the same biological sample. Experiment 3 was conducted on a biological independent sample.

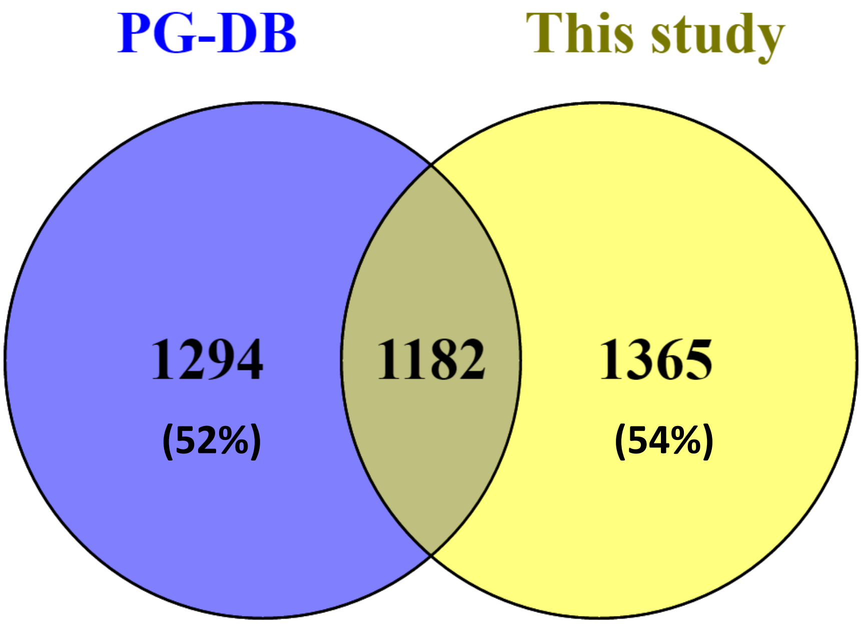

Supplemental Figure 2. Proteins identified in this study partially overlap with the proteins in the Rice Proteogenomics Database (Oryza PG-DB). Proteins with at least 2 peptides from Oryza PG-DB were compared to proteins identified by at least 2 unique peptides in this study. Percentages indicate the percentage of proteins unique to each dataset.

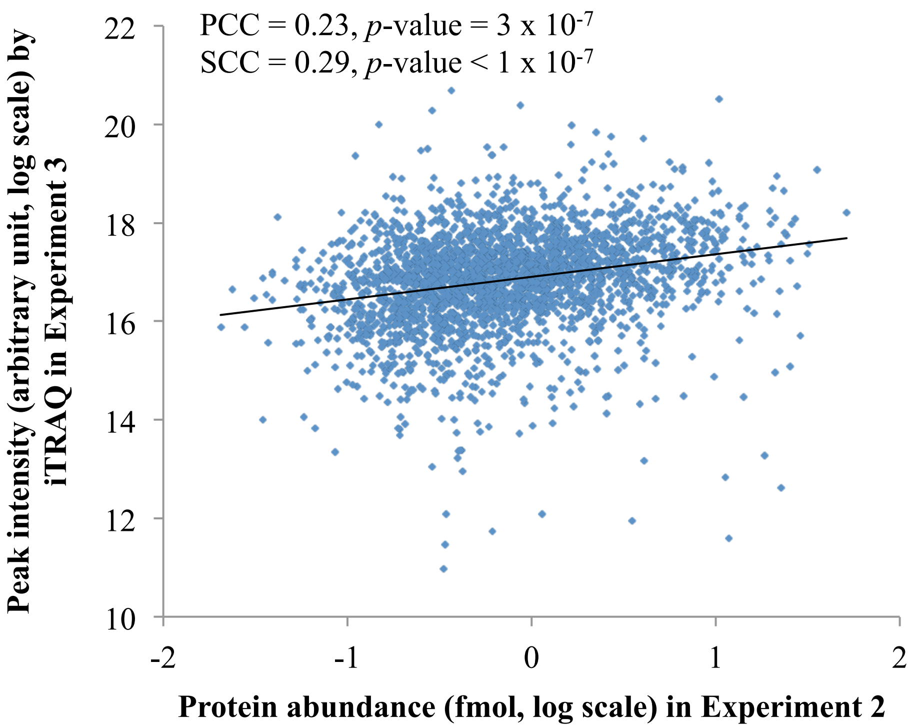

Supplemental Figure 3. Relative protein abundance as quantified by normalizing peptide counts in Experiment 2 and by isobaric tags for relative and absolute quantitation (iTRAQ) in Experiment 3. For Experiment 2 the protein amount (fmol) is calculated based on the response factor (ion intensity per fmol) of peptides from an alcohol dehydrogenase standard. For Experiment 3, 4-plex iTRAQ labeling was applied to each technical replicate. The protein abundance is the average of three technical replicates for Experiment 2 and four technical replicates for Experiment 3. PCC, Pearson's Correlation Coefficient. SCC, Spearman's Correlation Coefficient. The trend line is a linear regression: $y = 0.46x + 17$

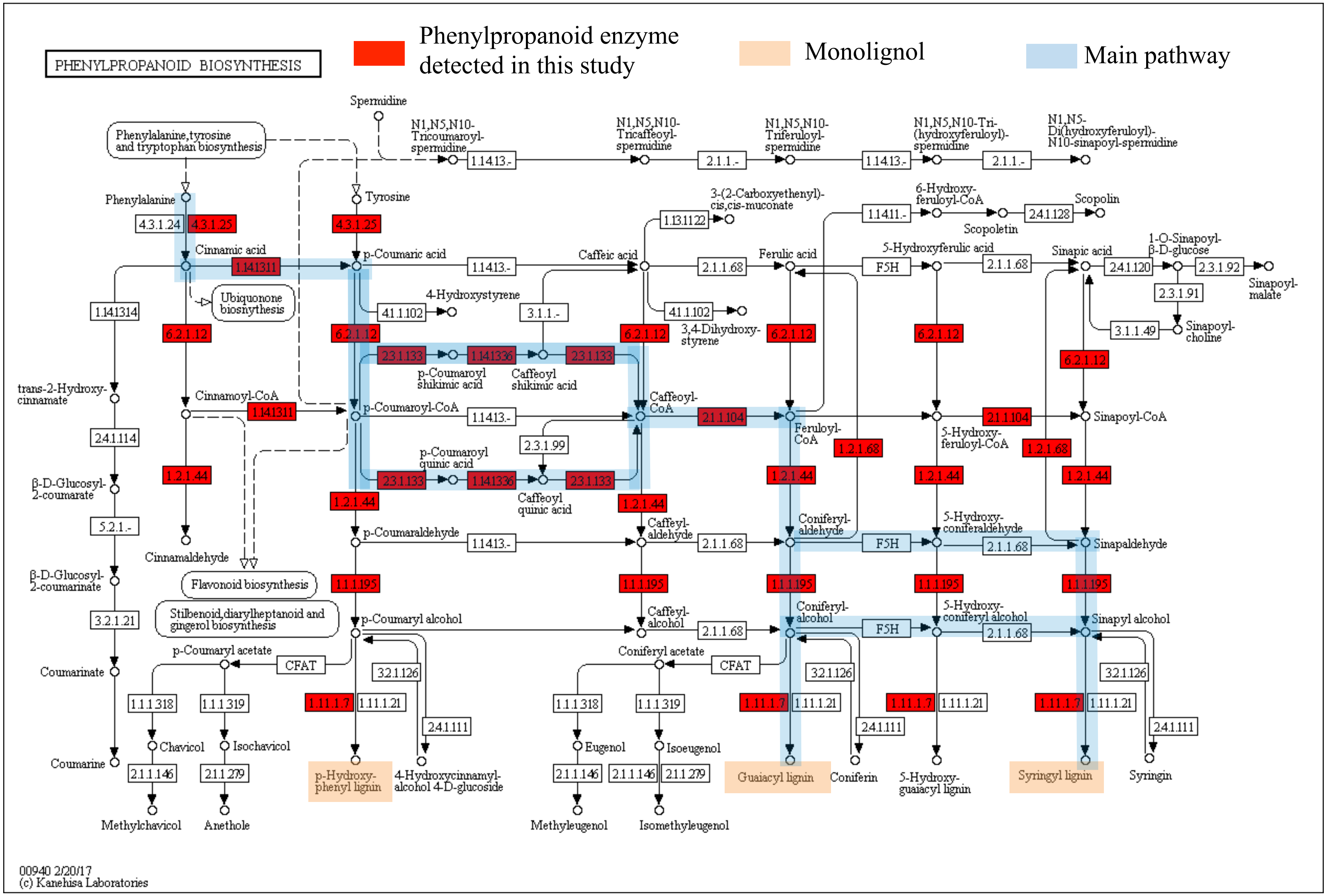

Supplemental Figure 4. Most phenylpropanoid biosynthesis enzymes were identified in rice elongating internode II samples. Identified proteins were mapped to the KEGG phenylpropanoid pathway (osa00940). Red boxes are phenylpropanoid synthesis enzymes identified in this study. Key monolignol synthesis reactions are indicated with blue shading. Orange shading indicates three common lignin subunits.

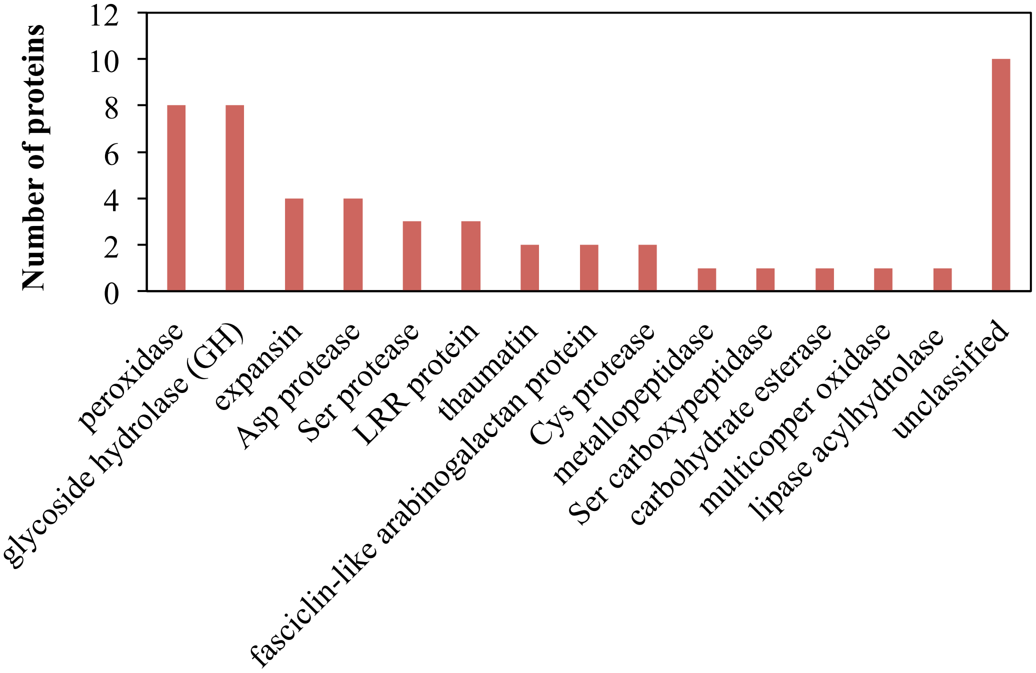

Supplemental Figure 5. Previously reported extracellular proteins identified in the rice elongating internode II. Proteins identified in this study were searched against the WallProtDB database and sorted based on molecular function.

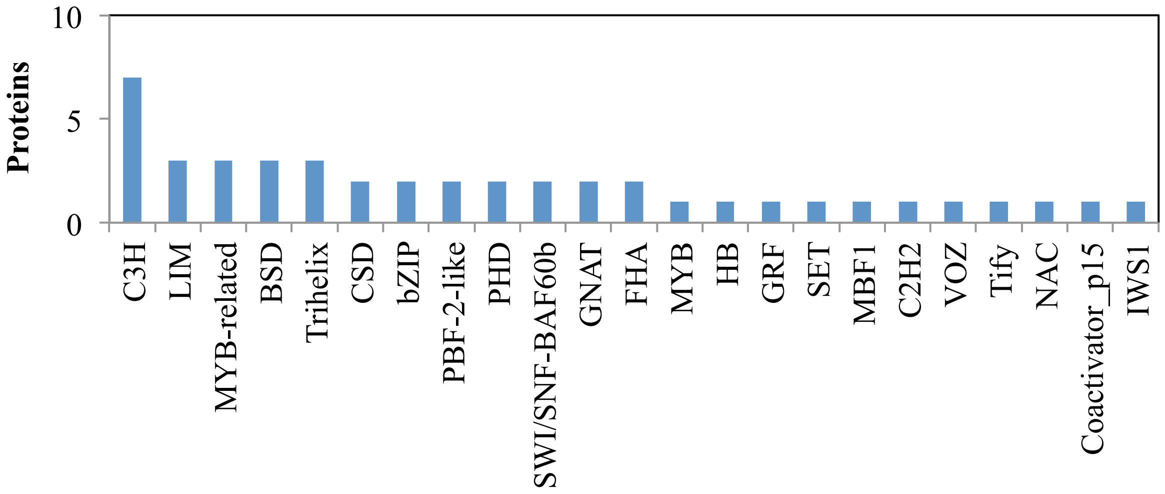

Supplemental Figure 6. Transcription factors identified in the rice elongating internode. Identified proteins were searched against a rice TF database and are sorted by transcription factor family.

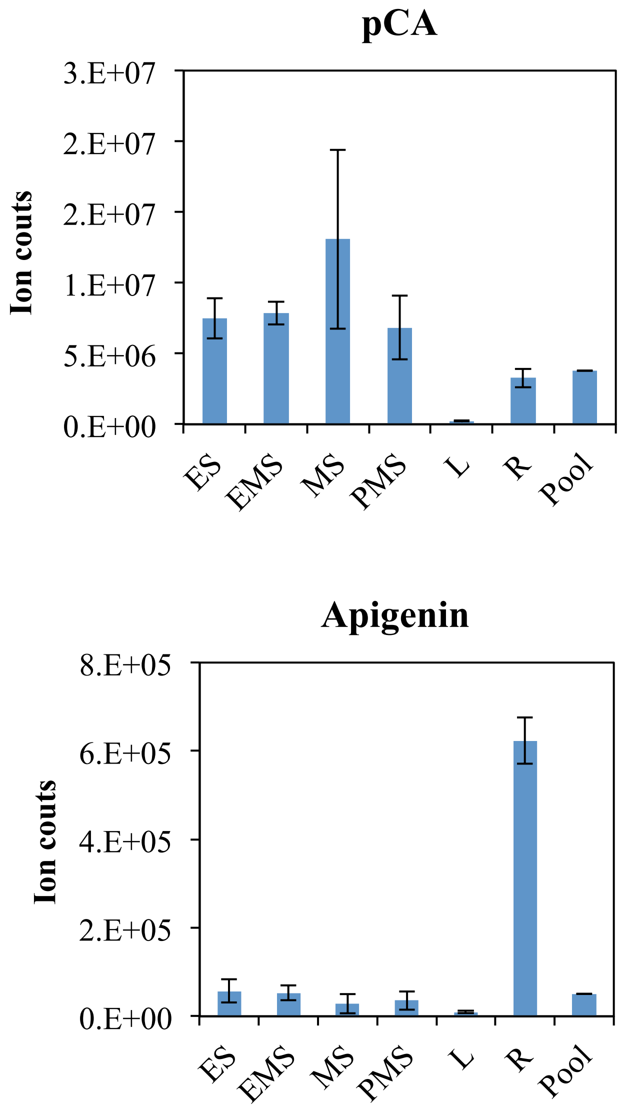

Supplemental Figure 7. The abundance of *p*-coumaric acid (*p*CA) and apigenin differ among organs. Error bars are standard deviation among three biological replicates, except for the pool, which had a single replicate.
